# Accuracy Improvement of In-line Near-Infrared Spectroscopic Moisture Monitoring in a Fluidized Bed Drying Process

**DOI:** 10.3389/fchem.2018.00388

**Published:** 2018-10-10

**Authors:** Andrey Bogomolov, Joachim Mannhardt, Oliver Heinzerling

**Affiliations:** ^1^Blue Ocean Nova GmbH, Aalen, Germany; ^2^Samara State Technical University, Samara, Russia; ^3^Drug Product Development, AbbVie Deutschland GmbH & Co. KG, Ludwigshafen am Rhein, Germany

**Keywords:** fluidized bed drying, moisture monitoring, NIR spectroscopy, light scatter, scatter correction, lighthouse probe, process analytical technology

## Abstract

An exploratory analysis of a large representative dataset obtained in a fluidized bed drying process of a pharmaceutical powder has revealed a significant correlation of spectral intensity with granulate humidity in the whole studied range of 1091.8–2106.5 nm. This effect was explained by the dependence of powder refractive properties, and hence light penetration depth, on the water content. The phenomenon exhibited a close spectral similarity to the well-known stochastic variation of spectral intensities caused by the process turbulence (the so-called “scatter effect”). Therefore, any traditional scatter-corrective preprocessing incidentally eliminates moisture-correlated variance from the data. To preserve this additional information for a more precise moisture calibration, a time-domain averaging of spectral variables has been suggested. Its application resulted in a distinct improvement of prediction accuracy, as compared to the scatter-corrected data. Further improvement of the model performance was achieved by the application of a dynamic focusing strategy when adjusting the model to a drying process stage. Probe fouling was shown to have a minor effect on prediction accuracy. The study resulted in a considerable reduction of the root-mean-square error of in-line moisture monitoring to 0.1%, which is close to the reference method's reproducibility and significantly better than previously reported results.

## Introduction

Fluidized bed drying is a common unit operation routinely performed in the pharmaceutical production of solid dosage forms. In a typical batch granulation process, the drying stage immediately follows either the fluidized bed or high-shear granulation stage. It is often considered as one of the most critical steps for achieving stable product quality, i.e., for obtaining granules with desired properties at their minimal variability. Therefore, a close monitoring of the residual moisture content in the process medium is necessary for any quality assurance system in granulate production.

In modern industrial practice, moisture is commonly analyzed in isolated samples. Karl Fischer titration is a classic water analysis technique that has been widely used for decades. A viable alternative accepted by pharmacopeias is thermogravimetric analysis with a drying balance that determines moisture content in the sample as percentage weight loss on drying (LOD). At present, both techniques are realized as compact desktop devices enabling the at-line analysis of samples taken from a running process.

For the process type studied here, the at-line analysis of granulate moisture content typically takes 20–30 min, representing a good alternative to off-line laboratory analysis of the final product. However, such operability is insufficient to carry out real-time process control, for example by generating alarms on abnormal process states and performing timely corrections. For the same reason, at-line analysis is hardly suitable for accurately determining the process end-point—the time point at which the product reaches its optimal properties. Therefore, instant in-line monitoring of the moisture content in fluidized bed drying is strongly desired to provide a necessary level of process control and to meet growing quality requirements.

Near-infrared (NIR) spectroscopy is an undoubted favorite among real-time sensor systems for moisture monitoring in the production of solids, specifically, in the drying step (Roggo et al., 2007; Burggraeve et al., 2013; Da Silva et al., 2014). In such systems, the diffuse reflectance spectra of the process material are typically measured through an immersion probe. The key advantages of NIR spectroscopy as an in-line analytical technique include the suitability for measurements in media of highly variable bulk density, nondestructiveness, and the capability to place the probe into an appropriate position within the process space while keeping it connected to a remote spectrometer through a fiber optic cable.

The classic NIR spectroscopic moisture analysis relies on two intensive water absorption bands around 1,440 and 1,930 nm, enabling quantitative determination of the moisture in a wide concentration range. In low-selective NIR spectra, the component bands are essentially overlapped and their quantitative analysis requires the application of multivariate modeling, also known as chemometrics. In particular, the partial least-squares (PLS) regression algorithm (Sjöström et al., 1983) is widely accepted in process chemometrics (Bogomolov, 2011).

Over the last decades, the practical acceptance of NIR spectroscopy for in-line moisture monitoring in fluidized bed processing of powders and solids have been constantly growing. Published works (Frake et al., 1997; Rantanen et al., 2000; Zhou et al., 2003; Green et al., 2005; Nieuwmeyer et al., 2007; Skibsted et al., 2007; Luukkonen et al., 2008; Mantanus et al., 2009; Alcalà et al., 2010; Corredor et al., 2011; Peinado et al., 2011; Burggraeve et al., 2012; Demers et al., 2012; Möltgen et al., 2012; Obregón et al., 2013) have focused on the general feasibility of the analysis or on the investigation of specific experimental or modeling aspects (e.g., important process influences, sampling, control strategy, and model transfer). At the same time, the resulting models are typically built and validated on relatively small sets of samples and batches, which can be accounted for by the technical complexity of industrial experiments. Hence, the accuracy estimates reported for similar process setups and conditions are very diverse (Zhou et al., 2003; Green et al., 2005; Nieuwmeyer et al., 2007; Skibsted et al., 2007; Mantanus et al., 2009; Alcalà et al., 2010; Corredor et al., 2011; Peinado et al., 2011; Burggraeve et al., 2012; Demers et al., 2012; Möltgen et al., 2012) and the “ultimate” moisture determination accuracy by in-line NIR spectroscopy under widely variable process conditions remains unknown. Therefore, despite significant progress, the method can hardly be regarded as completely established yet.

In-depth considerations of NIR spectroscopic analysis in terms of light propagation in the complex fluidized bed process medium are rare (Rantanen et al., 2000; Luukkonen et al., 2008; Burggraeve et al., 2013). One of the main obstacles complicating the NIR spectroscopic monitoring of fluidized bed drying is related to process turbulence. A highly variable density of the material around the probe, and consequently the quantity of light reaching the detector, causes intensive random fluctuations of the overall intensity of in-line spectra that are often referred to as the “scatter effect.” The problem is commonly resolved by preprocessing the spectra prior to the modeling step. The three most-used scatter correction methods are multiplicative scatter correction (MSC), standard normal variate (SNV), and spectral derivatives (Rinnan et al., 2009). The application of a scatter correction method to in-line process NIR spectra is ubiquitous; no exception has been found in the literature. In most cases, the choice of the preprocessing method is empirical or arbitrary.

In some publications, it was noticed that the NIR spectra expressed in the logarithmic reflectance units (lg(1/R)) exhibited a significant downward shift of the background as the drying progressed (Frake et al., 1997; Rantanen et al., 2000; Zhou et al., 2003; Luukkonen et al., 2008; Burggraeve et al., 2012). Two plausible explanations were suggested, both related to the altering of light scatter conditions in the course of drying. On one hand, the uniform decrease in spectral intensities could be caused by an increase in scattering particle size; this explanation was given by Burggraeve et al. (2012) and Frake et al. (1997). On the other hand, the presence of water on crystal surfaces affects the reflective properties of the granulated powder, resulting in a deeper light penetration and a subsequent higher absorbance of wetter samples (Rantanen et al., 2000; Luukkonen et al., 2008). Rantanen et al. (2000) provided an experimental evidence of the latter phenomenon by using the pharmaceutical excipient (microcrystalline cellulose) as well as inorganic glass beads (“ballotini”) with a known size distribution.

The present work aims at building an accurate and robust functional prediction model for in-line moisture content monitoring in fluidized bed drying based on a large representative set of designed process data. Both experimental and modeling factors have been scrutinized to improve the performance of the prediction model. A thorough exploratory data analysis has been applied to help understand the process multivariate trajectory delivered by in-line diffuse-reflectance NIR spectroscopy better. In this study, we focus on efficiently using of the whole spectral information, including both absorption and scatter-related effects of water, to improve the performance of in-line moisture monitoring.

## Materials and methods

Twenty-five pilot-scale fluidized bed drying batches of a pharmaceutical powder mixture were studied by using a 256-pixel diode-array TIDAS 1121 SSG NIR spectrophotometer with a wavelength range of 1091.8–2106.5 nm (J&M Analytik AG, Germany) that was equipped with the Lighthouse Probe™ (LHP) from GEA Pharma Systems nv – Collette, Belgium (Engler et al., 2009) immersed into the process medium. The LHP was periodically cleaned and recalibrated without process interruption (see section S1.4 of Supplementary Material). The total number of cleaning cycles in all batches was 19.

The data of each batch included from 396 to 1,213 NIR spectra collected at 5-s intervals (16,303 spectra in total). In the course of the process, 301 samples of about 5 g (between 5 and 26 samples from each batch) were isolated and analyzed for moisture content as weight loss on drying using a HR73 halogen moisture analyzer (Mettler Toledo GmbH, Switzerland). Reproducibility checks for three LOD analyzers performed during the whole study showed that the measurement standard deviation error does not exceed 0.06% (section S1.2 of Supplementary Material).

The main process and the sample information are summarized in Table S-1. Out of the 301 samples, three were rejected from further analysis as evident outliers (section S2.3.1 of Supplementary Material).

Individual batch conditions were set in accordance with a developed experimental design to cover the whole range of practical process variability. Moisture content in the selected samples varied between 2.38 and 25.92%. The active pharmaceutical ingredient (API) was present in four assay levels: 0 (placebo), 0.1, 1.0, and 10.0 mg. The range of process temperatures was 30.5–49.7°C. Eight batches (88 reference samples) formed a validation subset that was representative of the process conditions and used for model validation; the other 17 batches were used as the calibration set in that case (Table S-1).

A subset of 101 experimental samples were additionally analyzed off-line by using an MPA Fourier-transform (FT-) IR spectrometer (Bruker, Germany) with an integrating sphere (section S1.5 of Supplementary Material).

Principal component analysis (PCA) and PLS regression are multivariate data analysis algorithms described in the literature (Sjöström et al., 1983; Wold et al., 1987). The multivariate spaces, namely, PCA model principal components (PCs) and PLS latent variables (LVs) represented by their score (**t**) and loading (**p**) vectors, were used for exploratory data analysis. Conventional data preprocessing methods employed were MSC, SNV, and first-derivative using the Savitzky–Golay smoothing filter, as described by Rinnan et al. (2009).

Three validation techniques were applied with each regression model: leave-one(-sample)-out (LOO), a.k.a. full cross-validation (CV), leave-a-batch-out (LBO) CV, and validation by a preselected set (Table S-1). The performance of the models was characterized by root-mean-square errors (*RMSE*) of calibration, validation, and prediction, as well as corresponding determination coefficients *R*^2^.

A detailed description of data acquisition and analysis is given in section S1 of Supplementary Material.

## Results and discussion

### Exploratory analysis of in-line spectral data

**Figure 1:**
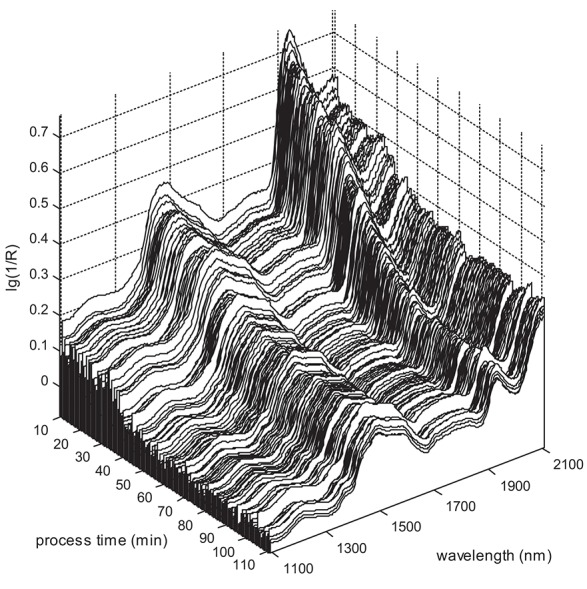
In-line NIR spectra in batch B03.

Figure 1 presents a set of 1,213 in-line NIR spectra obtained in batch B03 (Table S-1). An expected intensity reduction of the main water band in the 1,920–1,940 nm range during the process is clearly observed. Another distinct feature is the high variability of spectral intensities over the whole wavelength range (the so-called “scatter effect”), caused by strong instant density fluctuations of the granulate (and its spatial distribution) around the probe.

**Figure 2:**
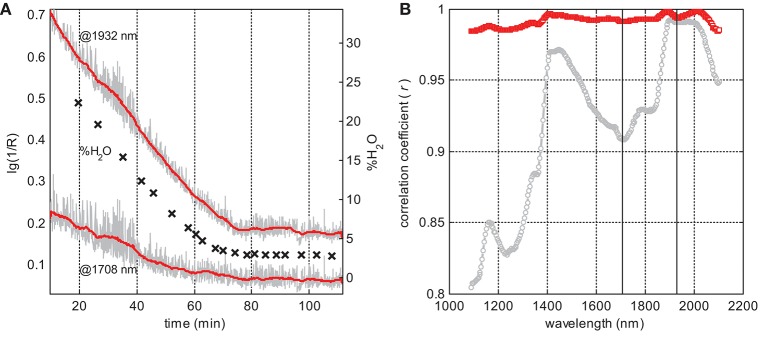
Exploratory analysis of B03 data: **(A)** raw (gray line) and smoothed (red line) spectral intensities at the two selected wavelengths and reference moisture content (crosses) vs. process time; and **(B)** correlation coefficients between the moisture content and spectral intensities at individual wavelengths for raw (gray circles) and smoothed (red squares) data; vertical lines at 1,708 and 1,932 nm correspond to the dependencies presented in **(A)**; data were smoothed with a 47-point window.

At the same time, the overall spectral intensity tends to fall gradually during the process, generally following the dynamics of water reduction. This trend can be illustrated by the time dependencies of the spectral intensity at two separate wavelengths: 1932.0 nm at the maximum of the main water band and 1708.1 nm where no noticeable water absorption is expected. Both intensities strongly correlate with the reference moisture content (Figure 2A). Data smoothing along the time scale makes this correlation even more distinct.

The moisture- and time-dependent changes in the batch processes can be effectively visualized by using data animation (section S2.1 and Video S-1, Supplementary Material). Animated spectral data reveal the same trends, namely water band reduction and stochastic background variation accompanied by a gradual fall of the spectrum intensity in the whole range.

In this situation, preprocessing is desirable, but it should be applied to the data variable vectors, i.e., along the time scale, as shown in Figure 2A. As the turbulence effect is supposed to be pure noise, the smoothing of variables is a straightforward way to eliminate it with a minimal loss of the informative variance.

One of the simplest smoothing techniques, the moving window averaging algorithm, has been used to preprocess the matrix of spectral data **X**. In this method, each element *x*_*ij*_ in **X**, where *i* and *j* are respectively the object (spectrum) and variable (wavelength) indices, is replaced by a corrected value $x_{ij}^{s}$ calculated as a mean of the surrounding points within a window having the width defined by an odd number *k* (Equation 1):

(1) $$x_{ij}^{s}=\frac{\sum_{i=i-(k-1)/2}^{i=i+(k-1)/2}x_{ij}}{k}$$

The transformation is performed for each variable in **X**. (*k* – 1)/2 end-points on each side of the variable vector were smoothed with a reduced window of (*l* – 1) ^.^ 2 + 1 points, where *l* is the point ordinal number from either spectrum end.

Data averaging within a selected time window is similar to a respective enhancement of the spectrum acquisition time, thus enlarging the virtual sample size captured by a single measurement. However, in contrast to the measurement time adjustment, the mathematical averaging does not place any limit on the time step of data acquisition, i.e., it can be performed with a time window that is much wider than the physical step size. A positive effect of the variable smoothing for the modeling of a fermentation process data has been reported (Skibsted et al., 2001).

Pair-wise correlations between the LOD values and the intensities at individual variables in the corresponding (closest to the sampling times) in-line spectra were analyzed in the whole wavelength range. Figure 2B presents linear correlation coefficients (*r*) as a function of wavelength in B03. All spectral variables exhibit a strong intensity correlation with the moisture content, even in the raw data. Eliminating the process noise using the suggested averaging method (Equation 1) results in a dramatic enhancement of *r*. It also looks natural that correlation maxima are observed around major water bands. However, even beyond the water absorbance regions, this correlation is very high. Thus, the lowest *r* observed in B03 at the short-wave end of the spectral region is still greater than 0.8 (Figure 2B); after the smoothing, this value increases to 0.98. Similar dependencies were observed for all the 25 studied batches.

A high correlation of lg(1/R) with the moisture content in the whole studied NIR range is in agreement with some published observations. This phenomenon can be explained by altering the refractive properties of the granulate (Rantanen et al., 2000). Indeed, in the course of drying, the liquid bridges holding the primary particles together (Burggraeve et al., 2013) are replaced by air. The crystal–air interface is characterized by a higher difference of refractive indices than the crystal–water pair. Thus, drying leads to a higher scatter—and hence an increased quantity of diffusely reflected light reaching the detector—that corresponds to a decrease in the spectral intensity expressed in absorbance type of units. For relatively large particles constituting the granules, this effect should be wavelength-independent. An intuitive illustration of the particle wetting effect and its uncomplicated explanation using the representative layer theory was given by Dahm (2013). A similar correlation of the Raman spectral background with the moisture content was observed in our earlier studies on pellet coating (Bogomolov et al., 2010) and granulation process monitoring (Bogomolov, 2011), and was also explained by the effect of moisture on the light propagation conditions in the process medium. Considering the strength of the spectrum variable correlation with the moisture content observed in the whole range of process conditions studied, an earlier explanation of the phenomenon in terms of changing particle size distribution during the drying course (Frake et al., 1997; Burggraeve et al., 2012) has not been confirmed. This hypothesis does not agree with the complex shape of the correlation curve in Figure 2B. Particle size distribution can be a minor water-correlated factor affecting the spectra of the drying process, though.

The effect of humidity on the light penetration depth in porous materials can be compared to the watermark technique commonly used for banknote authentication. The very name of watermarks comes from the visual similarity of paper thickness variation and its wetting effects, both resulting in a decrease in the back-scattered light. Darkening of wetted powders (e.g., sand) is another manifestation of the same phenomenon that is not limited to the visible light and should be inherent in any material with a highly developed surface. The spectral variance related to the changing refractive properties of the powder is also expected to be present in the in-line process spectra. However, being wavelength-independent, the moisture-related spectral changes are masked by the stochastic “scatter effect” and then eliminated by any scatter correction. Earlier studies on in-line moisture analysis by using NIR spectroscopy neither paid any significant attention to the analytical information hidden in the “watermarks” nor attempted to use it in the modeling.

A deeper insight into the data structure and its modification by adopting different preprocessing methods was obtained by the PCA of augmented process data (section S2.2 of Supplementary Material) that makes possible the investigation of process trajectories of individual batches in the same multivariate factor space.

**Figure 3:**
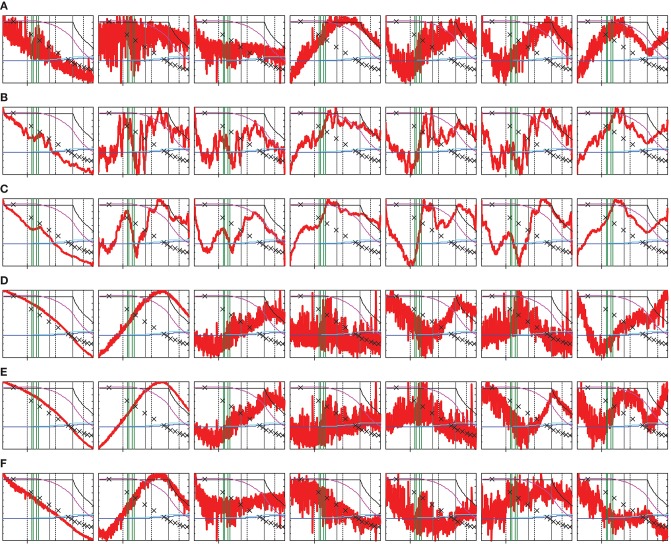
PCA scores (vertical axis, arbitrary units) vs. process time (horizontal axis, process time from 1,130 to 4,331 s, with the tick at 2,000 s) for batch B10. The plots in a line present individual scores **t**_1_-**t**_7_ (left to right) for different data preprocessing methods: **(A)** none; **(B,C)** variable smoothing with 15- and 47-point windows, respectively; **(D)** MSC; **(E)** SNV; and **(F)** first derivative using the Savitzky–Golay smoothing filter. Process parameters are shown overlaid: moisture content in reference samples (crosses); drying air temperature (black line), product and exhaust air temperatures (light and dark blue lines, respectively); exhaust air humidity (violet line); and LHP cleaning start/end points (vertical green lines).

As one can see from the scores of batch B10 taken as an example here (Figure 3 and Figure S-3), the first PC (95.49% of **X**-variance) of the raw-data model (Figure 3A) is strongly associated with the moisture content, while PC_2_ (4.23%) basically describes the process turbulence. A remarkable similarity of the first two loadings (Figure S-4a) with the correlation coefficient *r* = 0.998 is a confirmation of a close spectral affinity of these two phenomena. A scatter-driven correlation of spectral intensities with the moisture content is confirmed by the uniformly positive **p**_1_. A simultaneous presence of the water absorption peaks in this plot implies that PC_1_ tends to capture the whole variance due to the moisture reduction, related to both absorbance and scatter phenomena.

Although the process noise is basically described by PC_2_, it strongly pollutes PC_1_ and all further components in the raw-data model. The suggested smoothing method effectively eliminates this noise from the model scores (Figures 3B,C and Figures S-3b,c) without any essential change to the loadings (Figures S-4b,c). In contrast, the SNV, MSC, and first derivative (Figures S-4d-f)) strongly modify the whole factor space; they essentially remove random fluctuations from the first two score vectors (less noisy for the first derivative) but further PCs stay very noisy (Figures 3D–F). The smoothed data is suitable for exploring the process trajectories in the PCA factor space. Most of the minor features revealed in the refined scores **t**_2_-**t**_7_ (Figures 3B–F) can be assigned to certain process events, i.e., to changing process phases or LHP cleaning cycles. The PCA score plots for all batches can be found in Figure S-3.

**X**-variances captured by individual PCs (Table S-3 in section S2.2.2 of Supplementary Material) indicate at least six significant factors for all preprocessing methods, while the PC_8_–PC_10_ are definitely negligible. The PC_7_ seems to be a boundary case, and its significance should be proved by using other criteria. Considering spectrum-like loadings (Figure S-4) and process-reflecting scores, in particular in the time-wise averaged data (Figure 3C), seven PCs are likely to be relevant. Additional considerations helping to deduce a number of PCs in the augmented process data are considered in section S2.2 of Supplementary Material.

In general, the low variances captured by minor principal components PC_2_-PC_7_ (Table S-3) illustrate a much higher sensitivity of NIR spectroscopy to water than to other chemical or physical variability sources in the drying process medium. Nevertheless, a thorough study of the complete PCA model resulted in some practically important observations. Thus, LHP fouling and cleaning during the process has a minor effect on the observed in-line spectra, in particular, at the final process stage (section S2.2.1 of Supplementary Material).

An exploratory data analysis performed has revealed an essential correlation of all spectral variables with the moisture content. The PCA analysis of the united dataset (16,303 spectra) has shown that this effect is overlaid with a variation on the stochastic spectrum intensity caused by the process noise. Since both scatter-driven effects have similar spectral signatures, the application of conventional normalization or derivative preprocessing methods of scatter correction incidentally removes useful information contained in the spectrum background. Instead, it was suggested to perform the smoothing of spectral variables along the time domain, e.g., using a moving window average.

### Building an accurate PLS regression model of moisture content

For efficiently using the additional moisture-related information contained in the spectral variables, the dependence of model accuracy on averaging window width (WW = *k* points) has been studied. PLS models for all possible odd *k* values between 3 and 101 in different moisture ranges were compared (section S2.3.2 in Supplementary Material). Since the in-line smoothing of time dependencies results in a delay of 2*k* – 1 trajectory points (half WW) between the process and analysis times (Bogomolov, 2011), light smoothing is technically preferred. WW = 15 was found to be optimal in all cases as it provided an essential improvement of the model accuracy with a reasonable delay of 35 s. The full WW of 70 s approximately corresponds to a material circulation period in this process and dryer type. Thus, each portion of the granulate has a good chance of being exposed to spectroscopic measurement during this time. Due to the averaging, a virtual sample size captured by spectroscopic measurement, and hence the level of scrutiny of analysis, is extended. From this point of view, an optimal WW should correspond to an averaged spectrum that is representative of the bulk material volume, while remaining a nearly instant measurement compared to the total process time. This principle can be suggested as a rule of thumb for optimal data averaging in the drying process analysis and similar applications. A 47-point averaging was found to be a “global” optimum in our case; stronger smoothing does not lead to any significant gain. Based on these observations, 15- and 47-point smoothing windows have been chosen as benchmarks for model comparison (the respective preprocessing methods are designated as S15 and S47). Table 1 presents a summary of full-spectrum modeling results for different moisture ranges, preprocessing techniques, and validation methods.

**Table 1:** PLS regression statistics for in-line moisture content determination: model comparison for different moisture ranges and preprocessing techniques using different validation methods; all models were built with 7 LVs.

**Data^a^**	**nS^b^**	**PP^c^**	**Calibration**^d^		**LOO CV**^e^		**LBO CV**^f^		**Validation set**^g^	
			***RMSE***	***R*^2^**	***RMSE***	***R*^2^**	***RMSE***	***R*^2^**	***RMSE***	***R*^2^**
D	298	None	0.207	0.9981	0.222	0.9979	0.236	0.9976	0.246	0.9977
		S15^h^	0.181	0.9986	0.194	0.9984	0.209	0.9981	0.198	0.9984
		S47^i^	0.178	0.9986	0.191	0.9984	0.210	0.9981	0.197	0.9984
		MSC	0.264	0.9970	0.292	0.9963	0.341	0.9950	0.290	0.9969
		SNV	0.312	0.9958	0.342	0.9949	0.395	0.9933	0.334	0.9961
		1D2.15^j^	0.203	0.9982	0.221	0.9979	0.251	0.9973	0.256	0.9977
D_20_	289	None	0.190	0.9979	0.205	0.9976	0.216	0.9973	0.269	0.9967
		S15	0.169	0.9983	0.182	0.9981	0.195	0.9978	0.208	0.9979
		S47	0.166	0.9984	0.178	0.9982	0.190	0.9979	0.211	0.9979
D_15_	268	None	0.152	0.9978	0.161	0.9975	0.170	0.9973	0.146	0.9979
		S15	0.146	0.9980	0.155	0.9977	0.163	0.9975	0.137	0.9981
		S47	0.139	0.9982	0.147	0.9979	0.155	0.9977	0.129	0.9984
		MSC	0.175	0.9971	0.188	0.9967	0.210	0.9958	0.209	0.9963
		SNV	0.175	0.9971	0.191	0.9965	0.215	0.9957	0.184	0.9970
		1D2.15	0.153	0.9978	0.164	0.9974	0.181	0.9969	0.158	0.9976
D_10_	213	None	0.116	0.9967	0.124	0.9962	0.141	0.9951	0.129	0.9946
		S15	0.109	0.9971	0.116	0.9967	0.132	0.9957	0.122	0.9950
		S47	0.109	0.9971	0.116	0.9967	0.137	0.9954	0.121	0.9952

a Dataset used: D – full dataset, D_20_, D_15_, and D_10_ – datasets limited to LOD moisture content below 20, 15, and 10%, respectively;

b the number of samples without outliers (see section S2.3 of Supplementary Information);

c preprocessing applied;

d calibration statistics;

e full cross-validation statistics;

f leave-a-batch-out cross-validation statistics;

g validation set (Table 1) prediction statistics;

h variable averaging with 15-point window;

i variable averaging with 47-point window;

j *Savitzky–Golay first derivative with second-order polynomial and 15-point smoothing window*.

The data covers a wide range of moisture contents from 2 to 26% (Table S-1). As the prediction error may be nonuniform depending on the drying stages (Mantanus et al., 2009), several PLS models were built corresponding to moisture LOD ranges <20% (D_20_), <15% (D_15_), and <10% (D_10_), in addition to the full-data (D) models. The abundance of measurement points makes possible the use of this data reduction without a significant impact to the model quality. The upper value of moisture content noticeably reduces the *RMSE* (e.g., for LBO CV, it falls from 0.21 in D to 0.13 in D_10_), keeping *R*^2^ at the same high level of 0.997–0.998 (Table 1). A strong error dependence on the moisture content can be practically employed to improve the performance of moisture monitoring in general. Thus, prediction software can switch to a more precise model as soon as a certain moisture content level is reached, providing an automatic model “focusing” in the process course. By this way, the most critical final stage of drying can be monitored with the highest accuracy.

**Figure 4:**
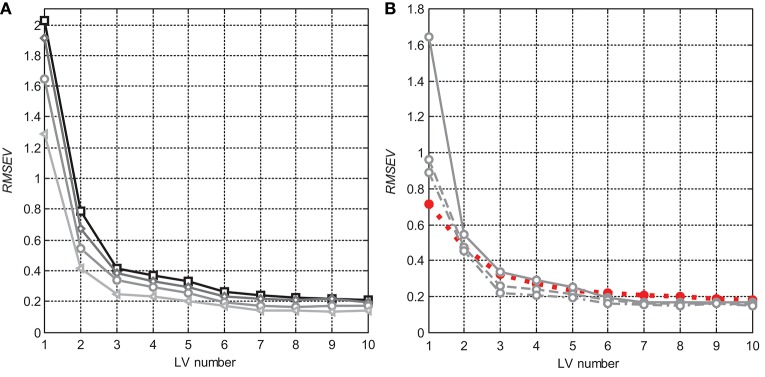
*RMSE* dependencies (LBO CV) on the number of LVs in PLS models: **(A)** for nonpreprocessed data in different moisture content ranges: D (squares), D_20_ (diamonds), D_15_ (circles), and D_10_ (triangles); and **(B)** D_15_ data with different smoothing degrees: none (solid), S15 (dashed), and S47 (dash-dotted), as well as for MSC preprocessing (red dotted, filled markers).

A number of LVs to be kept in PLS models was estimated from the *RMSE* of different validation methods and from the explained **X**- and **y**-variances (Table S-4). Figure 4 compares the LBO CV *RMSE* dependencies on the number of LVs for the models in different moisture ranges (Figure 4A) and data averaging degrees (Figure 4B). Their common trend is that the validation error reaches a plateau starting from the seventh LV; faint minima at higher factor numbers do not seem significant. Note that LBO CV is generally the most conservative (i.e., resulting in the highest errors) validation method in Table 1. Data scatter correction does not result in any model simplification as expected. Figure 4B shows that the validation *RMSE* for MSC-preprocessed D_15_ data is even higher than the *RMSEV* of the model obtained after moderate (S15) data smoothing. This effect is observed for any number of LVs higher than one. Starting from the sixth LV, the prediction error after MSC becomes even worse than in the raw-data model. This behavior agrees with the earlier PCA-based conclusion that conventional scatter correction refines only the two first factors of the multivariate space, transferring the process noise into higher yet significant model dimensions.

The analysis of the captured **X**- and **y**-variances (Table S-4) exhibited similar trends. It was also shown that seemingly insignificant variances captured by the seventh LV in the calibration data are still in agreement with the respective precisions of the NIR spectrometer and the LOD analyzer (section S2.3.3 of Supplementary Material).

The first two PLS loadings (Figure S-6) are almost identical to those in the augmented PCA (Figure S-4); therefore, both multivariate modeling spaces are essentially the same. Meaningful shapes of the first seven loadings, which are similar in PCA (Figure S-4) and PLS models (Figure S-6) as well as PCA scores (Figure 3), provide an additional justification of the chosen model's complexity. The noticeable positive offset of **p**_1_ in raw and smoothed data models (Figures S-6a-c) indicates that PLS regression makes use of both absorbance and scatter-correlated variances for moisture calibration. The loadings **p**_3_ to **p**_7_ still exhibit similar (as in PCA) interpretable spectrum-like features. Therefore, seven LVs were found to be optimal for all moisture ranges and data preprocessing methods, consistent with the earlier PCA result for all spectral data. This number is also reasonable, considering the physical and chemical complexity of the process as well as the anticipated nonlinearity of spectral responses. It is also acceptable from the point of view of calibration set size.

Table S-4 also confirms the efficiency of variable smoothing. Cumulative **y**-variances grow with the averaging WW, reducing a misbalance between the **X**- and **y**-variances for any number of LVs, in particular, for LV_1_. Starting from LV_3_, the **y**-variance captured in smoothed data becomes higher than that in the models preceded by scatter correction (e.g., MSC). In detail, the problem of deducing the optimal number of LVs is considered in section S2.3.3 (Supplementary Material).

**Figure 5:**
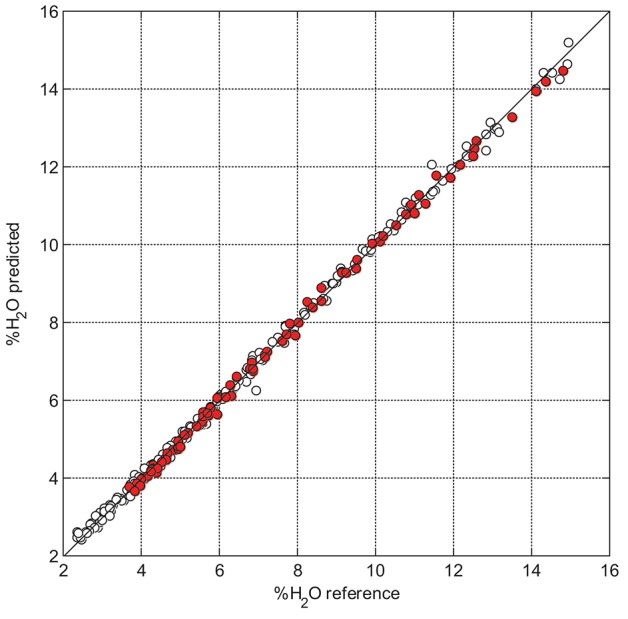
PLS predicted (7 LVs) vs. measured moisture content for D_15_ with 15-point smoothing; calibration and validation samples are presented by hollow and red-filled markers, respectively.

Validation statistics presented in Table 1 evidences that the suggested data averaging approach is advantageous as compared to the MSC, SNV, and first derivative using the Savitzky–Golay smoothing filter. It is remarkable that any scatter correction (most essentially, MSC or SNV) leads to higher calibration and validation errors than those for raw spectral data (This comparison is provided for D and D_15_, but it holds for all datasets). Figure 5 illustrates the model performance achieved in D_15_.

A subset of 101 process samples was additionally analyzed off-line by using a high-resolution FT-NIR spectrometer (section S2.3.5 of Supplementary Material). The integration sphere applied in this case excluded any scatter-related stochastic variation of spectral intensities. Nevertheless, all spectral variables (including the background signal) exhibited the same strong correlation with the sample moisture content (Figures S-9, S-10), as in the case of in-line spectra (Figure 2). This fact confirms our previously given explanation of this effect in terms of changing light propagation conditions. Moreover, the performance of the PLS model built on 96 off-line spectra (samples with LOD > 15% were used) was found to be essentially the same (cross-validation *RMSE* = 0.108) as in the model built on respective averaged in-line spectra (S15) of the same process samples (Table S-6). This remarkable result provides an additional confirmation of the efficiency of the suggested method. For more details on the off-line analysis results, see Supplementary Material, section S2.3.5.

The time dependencies of the predicted moisture content in B12 (Figure S-7) illustrate the additional advantages of the suggested preprocessing technique. Variable smoothing most efficiently eliminates the noise contained in process trajectories at the beginning of the drying process, when the moisture content is greater than 15%. It also helps avoid prediction artifacts related to probe cleaning during the “wet” process stage. Section S2.3.4 in Supplementary Material provides a detailed discussion of the predicted drying trajectories.

In numerous publications on in-line diffuse-reflectance NIR monitoring of fluidized bed drying and similar processes, data analysis is always prefaced by MSC, SNV, or derivatives without exception. A mandatory application of corrective preprocessing may only be justified in preliminary feasibility studies, when the small calibration/validation dataset does not allow for building models of adequate complexity. The results reported here could be used as evidence for the destructiveness of scatter correction for the moisture calibration, as it eliminates a significant portion of the useful variance. Similar ideas have been formulated in the literature (Chen and Thennadil, 2012), where the information content of MSC coefficients was analyzed. The PLS capability of employing the quantitative information delivered by the scatter has earlier been illustrated in other applications, in particular in particle size analysis (Nieuwmeyer et al., 2007) and the quantitative determination of fat and protein in milk (Bogomolov et al., 2012). In these cases, the predictive models built on raw data exhibited a noticeably better performance, as compared to those in which any scatter-correction was applied. For in-line process data, the suggested smoothing approach, performed in a time rather than spectral domain, presents a viable alternative to the classic scatter correction of spectra, to eliminate noise while preserving useful information contained in the spectral variables.

## Conclusions

In light of our presented results, the following recommendations to practical NIR spectroscopic monitoring of moisture content in fluidized bed drying and similar process types can be formulated. A very common practice of *a priori* scatter correction of in-line process spectra prior to the multivariate calibration is generally discouraged, because it may eliminate an essential part of the water-related variance from the data and thus deteriorate the resulting prediction model. To avoid this, quantitative modeling should be prefaced by an exploratory analysis of the raw data to investigate the relevance of both absorbance and scatter-related effects of moisture by using a sufficiently large representative set of designed samples and process conditions. These considerations are equally valid in cases when water content is not directly determined, but it should be taken into account by an accurate multivariate model as an important process factor. Process noise, i.e., stochastic background and intensity variations of in-line spectra, can be efficiently eliminated with a minimal loss of useful information by means of data smoothing along the time scale. The parameters of smoothing strengths should be adjusted depending on the process scale and dynamics. Building accurate quantitative models should rely on a methodically determined number of latent variables. A deliberate application of less LVs than their optimal number following from the model diagnostics—sometimes done by researchers to guarantee an avoidance of overfitting—is not always justified. An underfitting may often be more undesirable for model prediction accuracy.

## Author contributions

AB conceived and wrote the paper and analyzed the data. JM conceived the project and organized and planned industrial experiments. OH performed the experiments and analyzed the data.

### Conflict of interest statement

OH is an AbbVie employee and may own AbbVie stock/options. The remaining authors declare that the research was conducted in the absence of any commercial or financial relationships that could be construed as a potential conflict of interest.
