# Supplementary material for: Accuracy Improvement of In-line Near-Infrared Spectroscopic Moisture Monitoring in a Fluidized Bed Drying Process
Source: Front Chem. 2018 Oct 10;6:388. doi: 10.3389/fchem.2018.00388 (PMC6192013; doi:10.3389/fchem.2018.00388)
Supplement: Supplementary file 2 [file Data_Sheet_1.PDF]

SUPPORTING INFORMATION

Accuracy improvement of in-line near infrared spectroscopic moisture monitoring in a fluid bed drying process

OR (alternative title)

Use of "watermarks" in near infrared spectra to improve the accuracy of in-line moisture monitoring: A fluidized bed drying process study

Andrey Bogomolov*[1,2], Joachim Mannhardt[1] and Oliver Heinzerling[3]

[1] *Blue Ocean Nova, Anton-Huber-Straße 20, 73430 Aalen, Germany*

[2] *Samara State Technical University, Molodogvardeyskaya Street 244, 443100 Samara, Russia*

[3] *Drug Product Development, AbbVie Deutschland GmbH & Co. KG, Knollstraße 50, 67061 Ludwigshafen am Rhein, Germany***

***** Corresponding author: e-mail: ab@globalmodelling.com; formerly: J&M Analytik AG, 8 Willy-Messerschmitt-Straße, 73457 Essingen, Germany

** Formerly: Solvay Pharmaceuticals, Weesp, The Netherlands

ABSTRACT: This supplementary information document contains additional experimental details, results, discussions, and conclusions referenced from the main paper "Accuracy improvement of in-line near infrared spectroscopic moisture monitoring in a fluid bed drying process". Some elements of the present document have incompatible formats, and thus, are provided as separate files: Video 1.avi (Video S-1; a video clip); Image 1.pdf (Figure S-3; a large-size figure); Image 2.pdf (Fig. S-8; a large-size figure); their captions are given in the text of the present supplementary information file.

S1. Additional experimental details

S1.1. Granulation and drying

Fluidized bed drying process studied here followed the high-shear granulation of a pharmaceutical powder mixture. At the granulation stage 14 kg of microcrystalline cellulose PH102 (FMC Europe BV, Brussels, Belgium), 675 g of povidone, and 150 g of crosspovidone were granulated in a high shear mixer (Gral 75, GEA Pharma Systems nv – Collette, Wommelgem, Belgium) for 5 min (impeller speed 1, chopper speed 0). Aqueous solution (5.75 kg) of a non-disclosed active pharmaceutical ingredient (API) in demineralized water was used as a binder and added during the first minute of the granulation. After process completion, the wet granulate was transferred to a fluidized bed dryer T/GS2-K2-F1 (GEA Aeromatic Fielder, Bubendorf, Switzerland) via a vacuum transport. Before being charged, the fluidized bed was preheated to 50°C with an inlet air flow of 300 m^3/h. During the drying the air flow was kept constant for each process phase, whereas the inlet temperature control was used to adjust a desired product temperature.

Data from 25 designed drying batches was collected over 15 experimental days. Process parameters were systematically varied to train the model working in a wide range of possible production conditions. Drying air temperature was changing between 90°C and 40°C. At the initial process phase of the most active drying it was set to 90° and then manually reduced. The air flow rate was varied between 250 and 300 m^3/h. Actual inlet, outlet, and product temperatures as well as relative humidity of the inlet and outlet air were continuously monitored during the drying process. Four API assay values were applied. The main process and sample information is summarized in Table S-1. Calibration/validation subsets were defined to be representative of the whole process parameters, but the marginal values (bold numbers in Table S-1) were avoided in the validation samples.

Table S-1. Process data description.

ID[a]	Day[b]	nSp[c]	nS[d]	nO[e]	Set[f]	API, mg	ST[g], s	ET[h], s	nC[i]	%LOD		Prod. t °C[j]	
										min	max	min	max
B01	1	841	11	0	val	0.1	701	4899	0	4.23	21.41	31.6	38.3
B02	2	831	11	0	cal	0.1	392	4579	1	3.82	**25.92**	**30.5**	38.2
B03	2	1213	20	0	cal	0.1	571	6692	2	2.73	22.38	31.2	**45.3**
B04	3	640	7	0	val	0.1	526	3751	1	5.95	21.49	31.6	38.2
B05	3	589	6	0	cal	0.1	1921	4934	1	5.09	16.29	31.7	38.3
B06	3	537	7	0	cal	0.1	1026	3738	1	6.34	22.38	**30.7**	38.1
B07	4	842	10	0	cal	0.1	996	5231	1	2.70	13.05	32.7	**45.0**
B08	4	552	8	0	cal	0.1	952	3704	0	5.57	22.38	30.8	37.7
B09	5	498	6	0	cal	1.0	1855	4338	0	5.82	**23.05**	32.1	38.2
B10	5	642	12	6	cal	1.0	1130	4331	0	4.89	**22.72**	32.5	38.4
B11	6	641	8	3	cal	1.0	1682	4998	2	4.36	16.25	32.7	38.2
B12	7	835	18	9	val	1.0	875	5175	2	3.81	16.88	32.1	38.3
B13	7	638	9	3	cal	1.0	560	3903	1	5.12	20.37	32.1	38.2
B14	8	591	14	6	cal	1.0	5805	8847	1	3.03	14.54	31.1	38.2
B15	9	537	6	2	cal	1.0	1517	4195	0	5.59	14.97	31.5	38.3
B16	10	548	6	2	val	1.0	100	2836	0	5.59	11.57	32.6	38.5
B17	11	403	9	4	cal	10.0	1271	3351	1	5.76	16.68	**30.7**	37.1
B18	12	524	5	2	val	10.0	879	3495	0	5.76	11.11	33.9	38.3
B19	12	492	10	4	cal	10.0	3382	5834	0	4.81	17.43	32.2	38.3
B20	13	497	9	4	val	10.0	2109	4591	0	3.80	9.92	34.1	37.9
B21	13	667	17	9	val	10.0	663	3995	0	4.26	19.07	32.6	38.3
B22	13	610	24	15	val	10.0	493	3539	0	4.23	20.97	32.6	38.6
B23	14	1098	26	8	cal	10.0	179	5931	**4**	**2.44**	21.06	32.8	**49.7**
B24	14	641	24	13	cal	10.0	148	3459	**1**	**2.38**	15.01	33.7	**49.7**
B25	15	396	18	11	cal	**0**	0	1975	0	4.25	11.02	31.7	36.9
**Total[k]**	**15**	**16303**	**301**	**101**	**88[l]**	**0-10**	**-**	**-**	**19**	**2.38**	**25.92**	**30.7**	**49.7**

[a] Batch ID; [b] experimental day; [c] the number of spectra; [d] the number of reference samples; [e] the number of samples analyzed off-line; [f] belonging to calibration or validation set; [g] measurement start time with regard to the process time; [h] measurement end time with regard to the process time; [i] the number of probe cleaning cycles; [j] product temperature; [k] summary for the whole data set: the total numbers, intervals or maximal/minimal values; [l] the number of samples in the validation set.

S1.2. Sampling and moisture analysis

Samples were isolated from the fluidized bed using a built-in manual sampler. The moisture content was determined with a halogen drying balance HR73 (Mettler Toledo GmbH, Griefensee, Switzerland) as the weight percent loss on drying (LOD). About 5 g of each sample was taken for the LOD analysis. The halogen balance temperature was set to 105°C and the drying rate of 1 mg/50 s was chosen as a switch-off criterion. Reproducibility of three analyzers used for the measurements were characterized by the mean and standard deviation LOD values calculated from eight independent process samples (about 5 g each). The results are presented in Table S-2.

Table S-2. Reproducibility of drying balance analyzers.

**No**[a]	**Analyzer 1**[b] 114555[c] 1118492515[d]	**Analyzer 2** 119570 1117373354	**Analyzer 3** 110731 1117473999
**1**	3.77[e]	3.87	3.96
**2**	3.83	3.93	3.93
**3**	3.85	3.93	3.98
**4**	3.82	3.97	3.94
**5**	3.85	3.90	3.95
**6**	3.92	3.95	3.97
**7**	3.91	3.86	3.99
**8**	3.86	3.80	3.92
**Mean**[f]	3.85	3.90	3.96
**STD**[g]	0.05	0.06	0.02

[a] Sample (measurement) ordinal number; [b] thermogravimetric drying balance device number; [c] producer's SAP number; [d] producer's SNR identification number; [e] per cent mass loss on drying (LOD); [f] mean LOD value for an analyzer; [g] standard deviation of LOD value for an analyzer.

S1.3. In-line NIR spectroscopic measurements

In-line spectra were acquired with TIDAS 1121 SSG NIR spectrophotometer (J&M Analytik AG, Essingen, Germany) having a 256-pixel diode-array detector with the working range of 1091.8–2106.5 nm. Process spectra were collected every 5 s with the integration time of 70 ms at 20 accumulations (the total acquisition time of 1.4 s) through a Lighthouse Probe™ (LHP) by GEA Pharma Systems nv – Collette (Wommelgem, Belgium) [M. Engler, A. Bogomolov, J. Mannhardt, Die Lighthouse-Probe, eine neuartige Sonde für die Prozessanalytik, Chemie Ingenieur Technik - CHEM-ING-TECH. 01/2009; 81(8):1114–1115]. The probe was mounted into the dryer at about the same height as the manual sampler. LHP was supplied with an automated cleaning system enabling full cleaning of probe's measurement head without

process interruption (section S1.4). In this study the probe cleaning was performed irregularly for selected batches (Table S-1) and different process stages, to analyze its effect on the spectra and models.

S1.4. LHP construction and operating phases

Lighthouse Probe^{TM} (LHP) is an immersion probe with seven radial sapphire windows that irradiate and receive back-scattered light enabling spectral measurements through 360°. The captured light is delivered to a remote spectrophotometer through a fiber optical cable. LHP is supplied with an automated cleaning system, which enables cleaning of the probe's measurement head without process interruption. The cleaning phase is followed by the probe drying and recalibration, *i.e.* reference spectrum renewal against a built-in standard. The total cleaning cycle usually takes 0.5 to 2 min. The probe's functional principle and its four operating phases are presented in detail in Fig. S-1.

To perform the cleaning the probe's head is pulled back from the measurement position (Fig. S-1a) into the cleaning position (Fig. S-1b), where the optical windows are flushed with the water or an appropriate cleaning fluid. Tap water was used for cleaning in the present study. The wash is followed by drying with an air flow, using the same pair of pipe connectors. Calibration step (Fig. S-1c) is performed whether independently or immediately after the cleaning. During the calibration, the reference spectrum is replaced by a new one, obtained using a built-in ring of standard reflecting material. The old and the new reference spectra are compared to check the effectiveness of cleaning. The backmost position of the head (Fig. S-1d) is necessary to perform the final cleaning of the entire drying bowl (clean in place or CIP procedure). During CIP both pipe connectors are typically used to force the compressed air, thus preventing a penetration of fluids into the compartment. The probe was operated by NovaPAC software (Expo Technologies LLC, Saint Louis, MO, USA). The same software was used to acquire NIR spectra during the process.

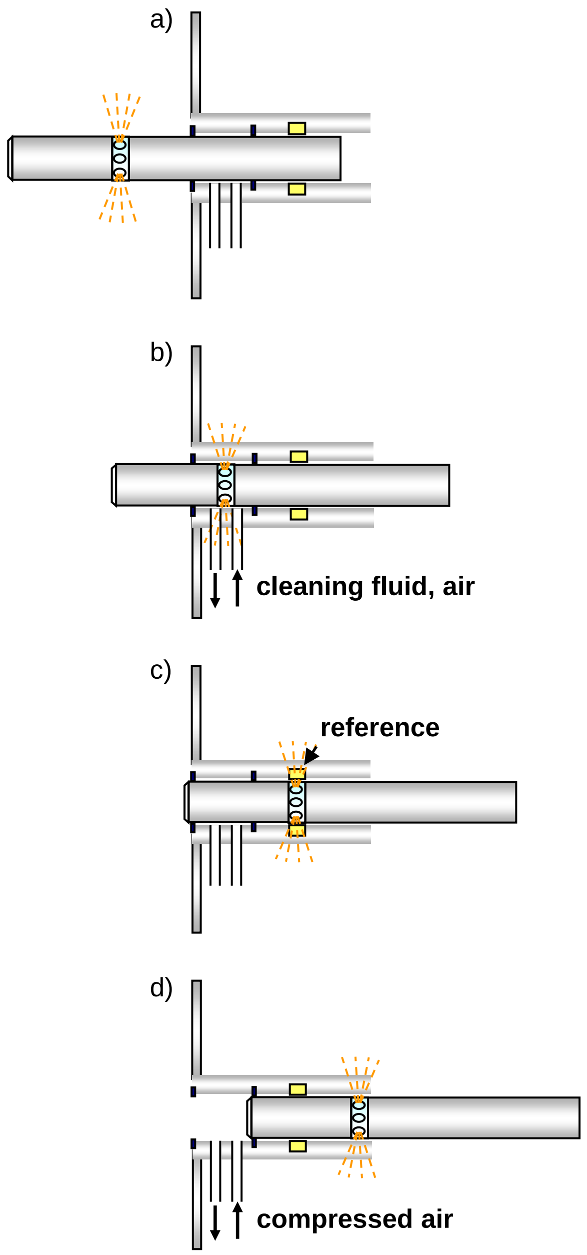

Fig. S-1. Lighthouse Probe^{TM} functional principle: a) measurement; b) cleaning and subsequent drying; c) cleanness check and re-calibration; and d) clean in place (CIP).

S1.5. Off-line NIR spectroscopic analysis

A Bruker MPA Fourier Transform (FT-)NIR spectrophotometer (Bruker Optik GmbH, Ettingen, Germany) was used for diffuse reflectance off-line NIR measurements. Spectra in the range of 12500–3600cm^{-1} were recorded with the resolution of 8 cm^{-1} at 32 scans per spectrum using an integrating sphere. Approximately 2 g of each sample were measured using a disposable injection vial.

For the purpose of comparison, the data analysis was performed on truncated FT-NIR spectra covering the region 1092.1–2106.1 nm (9156.7–4748.0 cm^{-1}). The truncation region was limited by the points closest to the borders of in-line spectrum range (1091.8–2106.5 nm).

S1.6. Data analysis software

The data analysis was performed in Matlab v7.7 (The MathWorks™ Inc., Natick, MA, USA) using PLS_Toolbox v.5.x-7.x by Eigenvector Research Inc. (Wenatchee, WA, USA).

S2. Additional results and discussion

S2.1. Exploratory analysis of individual batches using an animation technique

The moisture- and hence time-dependent changes in the batch process spectra can be effectively visualized using the following animation technique. Images of sequential in-line spectra are played back as movie frames; rates about twenty frames per second or higher provide a smooth playback. An example of such data movie including both raw and smoothed spectra in the batch B02 is provided in Video S-1.

[see “Video 1.avi” file]

Video S-1. Animated spectra of batch B02: grey – all spectra; black – current spectrum; red – current spectrum in the dataset averaged with a 15-point window (70 s).

Animated spectral data clearly reveal the same main trends as discussed in the manuscript: reduction of the water absorbance band and stochastic spectral variation that is accompanied by a gradual intensity fall in the whole wavelength range. These observations were found to be characteristic of all studied batches. Smoothing of spectral variables along the time scale, as suggested in section 3.1 of the paper, essentially eliminates the random variance from the data and reveals a wide-range correlation of the spectral intensity with the moisture content that is irrelative to the water absorbance bands. The magnitude of the whole-range moisture effect on the spectral intensity is comparable to the reduction of the main water band around 1940 nm. Therefore, it should be taken into account for an accurate quantitative modeling.

B02 spectral data averaged by a moving window with the width of 15 time points, which corresponds to the process time interval of 70 s, is presented by a red spectrum in the animated process movie. As a result of this time-domain smoothing (no traditional spectrum smoothing is

applied in this study!), the process noise is basically removed and both effects of the drying (absorbance- and scatter-related variances) become perfectly observed in the animated spectral data.

S2.2. PCA on the augmented spectral data

Further to the visual inspection of individual process runs, PCA was performed on the united set of in-line spectra from all 25 batches augmented with the common wavelength scale. Process trajectories revealed by PCA scores and loadings can be helpful to explain spectral data in connection with the process parameters and to estimate the effects of different preprocessing methods. Due to the augmentation, PCA produces a set of common loadings, whereas the resulting de-augmented scores keep their assignment to individual batches (Fig. S-2). PCA was performed on the augmented spectral data of all 25 batches, *i.e.* concatenated with a common wavelength scale, thus forming a single matrix with 16303 objects (spectra) and 256 variables. Time dependences of the first seven PCA scores for different preprocessing methods are presented in Fig. 3 of the paper (batch B10) and Fig. S-3 (for all batches). The respective common loadings are shown in Fig. S-4.

In the raw-data model, the first PC (95.49% of **X**-variance) is strongly associated with the moisture content, and PC2 (4.23%) basically describes the turbulence. This conclusion is based on the score shapes (Fig. 3a) and confirmed by the corresponding loadings (Fig. S-4). Remarkable shape similarity of the first two loadings (*r*=0.998) is an illustration of close spectral affinity of these two phenomena, as discussed in section 3.1. Beside the maxima at the water absorption wavelengths, $\mathbf{p}_1$ has a baseline that is uniformly positive in the whole spectral range (first plot in Fig. S-3a-c), which confirms the significance of the scatter-driven correlation of spectral intensities with the moisture content. Although the noise related to the process dynamics is essentially described by PC2 (Fig. 3a and S-3a), it strongly pollutes PC1 and all further PCs in the raw-data model.

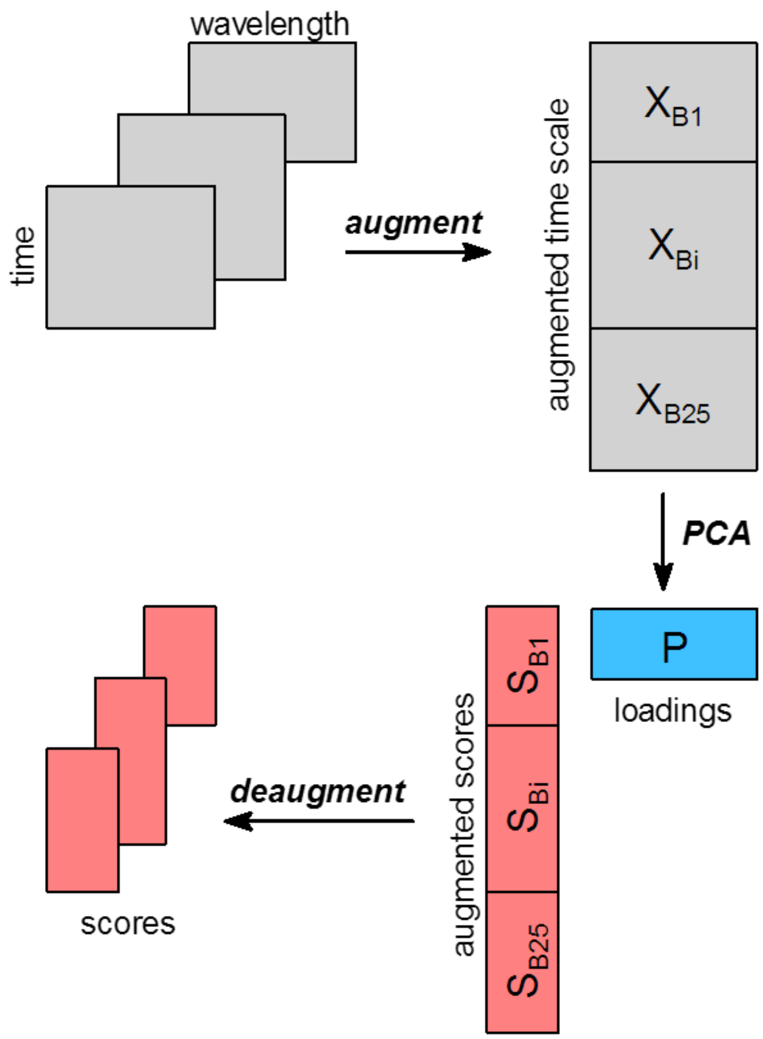

Fig. S-2. Augmented PCA schematic.

[see “Image 1.pdf” file]

Fig. S-3. PCA scores (vertical axis, arbitrary units) $\mathbf{t}_1$-$\mathbf{t}_7$ (left to right) versus process time (horizontal axis; the tick at 2000 s) for all batches (butch numbers are indicated from the left) for different data preprocessing methods: (a) none; (b), (c) variable smoothing with a 15- and a 47-point window, respectively; (d) MSC; (e) SNV; and (f) 1st derivative by Savitzky-Golay. Process parameters are shown overlaid: moisture content in reference samples (crosses), drying air temperature (black line) product and exhaust air temperatures (light and dark blue lines, respectively); exhaust air humidity (violet line) and LHP cleaning start/end points (vertical green lines).

Suggested smoothing method effectively eliminates this noise from the model (Fig. 3a-c and Fig. S-3a-c) without any essential changes of the respective loadings (Fig. S-4a-c). In contrast,

other preprocessing methods (Fig. S-4d-f and Fig. S-3d-f) dramatically modify the whole factor space; they essentially remove random fluctuations from the first two score vectors (this effect is smaller for the 1st derivative transformation), but further PCs stay extremely noisy. In all the models prefaced by a conventional scatter correction (Fig. S-3d-f) the first two score vectors are similar: $\mathbf{t}_1$ correlates well with the moisture content with some systematic deviation toward the lower values. The bell-shaped $\mathbf{t}_2$ that is common for MSC- or SVN-normalized data is characteristic of the preprocessing itself [Y.-C. Chen, S. N. Thennadil, Insights into information contained in multiplicative scatter correction parameters and the potential for estimating particle size from these parameters, Analytica Chimica Acta 746 (2012) 37–46], rather than of the process course.

Smoothed data suits well to explore process trajectories in the PCA factor space (Fig. 3c and S-3c). Most of the minor features revealed by the scores $\mathbf{t}_2$-$\mathbf{t}_7$ can be assigned to certain process events. Thus, sharp turns in the score linear plots tend to coincide with a process phase change or with an LHP cleaning cycle. Those effects can also be found in the scores resulting from the raw data or for preprocessing methods other than smoothing (Fig. 3a, d-e and S-3a, d-e). But their interpretation is complicated by the noise and some minor features get entirely lost.

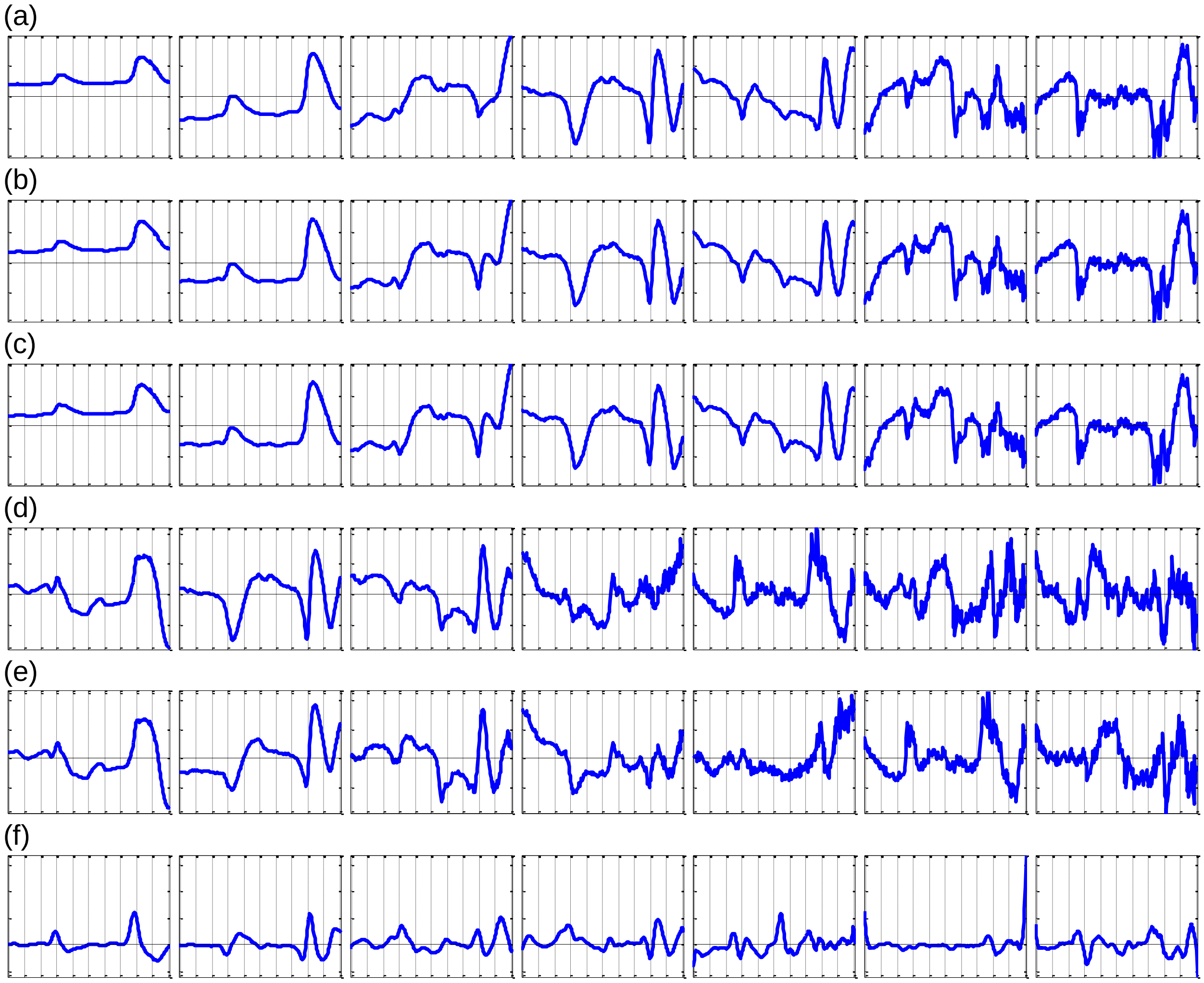

Fig. S-4. Augmented PCA of in-line spectral data: loading line plots versus wavelength ($\mathbf{p}_1$-$\mathbf{p}_7$, left to right) for different data preprocessing methods: (a) none; (b) and (c) variable smoothing with a 15- and a 47-point window: S15 and S47, respectively; (d) MSC; (e) SNV; and (f) first derivative by Savitzky-Golay. All loadings of the same model ($\mathbf{p}_1$-$\mathbf{p}_7$) have the same y-scales.

S2.2.1. LHP fouling and cleaning effects

Refined scores of the studied process batches provide an insight onto the well-known problem of probe window fouling that is commonly considered one of the main factors hindering diffuse reflectance NIR spectroscopy in accurate moisture monitoring in-line, *e.g.* [J. Mantanus, E. Ziémons, P. Lebrun, E. Rozet, R. Klinkenberg, B. Streel, B. Evrard, Ph. Hubert, Moisture content determination of pharmaceutical pellets by near infrared spectroscopy: Method development and validation, Analytica Chimica Acta 642 (2009) 186–192]. Indeed, at the initial process stage, as the granulate is wet and sticky, LHP cleaning actions (Fig. S-3c) result in noticeable corrective bends in the multivariate trajectory. Typically, it affects only minor scores $\mathbf{t}_3$-$\mathbf{t}_5$ (Fig. S-2), but in some cases may reveal itself even in the first PC, *e.g.* in batch B10 (Fig. 3

in the paper). After the cleaning, the moisture-correlated vector $\mathbf{t}_1$ in B10 exhibits a noticeable raise that is in agreement with some growth in spectral absorbance, as one can expect.

In spite of some trajectory deviation introduced by the fouling, the probe stays capable of following the moisture content. This fact can be explained by good penetration ability of the NIR light through the stuck granulate; LHP's seven optical ports strengthen its detection efficacy. Besides, moisture content in the crust surrounding the probe should follow (with some time delay) the process course due to the equilibrium with bulk process material. PCA-trajectories of studied batches (Fig. S-3) show that during the "wet" beginning stage of fluidized bed drying the probe cleaning is critical for accurate process monitoring. As the granulate reaches the moisture content of 10-15%, the cleaning has almost no effect any more. Typically, the crust falls out at that stage, while some inevitable probe contamination with dry product dust is negligible.

S2.2.2. Investigation of X-variances captured by individual PCs

X-variances captured by individual PCA factors (Table S-3) provide a deeper understanding of effects of different preprocessing methods on the data information content and help to deduce the optimal number of principal components to be considered in a PCA model.

Variable smoothing removes a noticeable part of the total data variance. At a 47-point window this reduction reaches more than 12%, *i.e.* $4.26 \cdot 10^{-3}$ versus $4.85 \cdot 10^{-3}$ (Table S-3), which can be taken as a rough estimate of stochastic variance share in the raw process data. The averaging tends to emphasize the first PC (**X**-variance adds almost 3% for S47) at the expense of PC2 and further PCA factors, where the variance reduction is accounted for by the noise elimination (Fig. S-3c). The refined scores in the smoothed data based models get higher contrast basically keeping their initial shapes (Fig. S-3b,c). Conventional preprocessing methods result in similar percent variances of PC1 and PC2, as in the raw and smoothed data cases, but further PCs exhibit much higher values, which is in agreement with their higher noise content.

X-variances captured by individual PCs (Table S-3) undoubtedly indicate six significant factors independently on the preprocessing applied, while the PCs eight to ten are negligible. The seventh PC represents a boundary case, and its significance should be proved using other criteria. Considering spectrum-like loadings (Fig. S-4) and process-related score shapes, specifically, in the time-averaged data (Fig. S-3b and c), one can suggest the relevance of all seven PCs. Generally low shares of the variance captured by the minor principal components PC2-PC7 (Table 2) illustrate much higher sensitivity of NIR spectroscopy to the water than to other chemical or physical variability sources in the drying process medium.

In order to estimate the practical significance of spectral information captured by the model factors, variable standard deviations corresponding to individual PCs (instead of their variances) can be used. Their values are helpful to justify the chosen model complexity. Being expressed in spectral intensity units, the standard deviation is directly comparable to the measurement precision (if the respective models are based on absorbance spectra, raw or smoothed). Thus, the maximum standard deviation among the variables in the reproduced data portion captured by PC7 ($\mathbf{t}_7$*$\mathbf{p}_7^T$) is above 0.001 (both in the raw- and smoothed data), which is certainly above the measurement precision of the used spectrophotometer type.

Table S-3. **X**-variances in augmented PCA models for different data preprocessing methods.

Method	V_0[a]	V_1[b]	V_2	V_3	V_4	V_5	V_6	V_7	V_8	V_9	V_{10}
**none**	4.85e-03	95.488	4.234	0.168	0.074	0.020	0.008	0.003	0.002	0.001	0.000
**S15**[c]	4.30e-03	97.887	1.862	0.142	0.075	0.017	0.009	0.003	0.002	0.001	0.001
**S47**[c]	4.26e-03	98.086	1.676	0.133	0.072	0.015	0.009	0.003	0.002	0.001	0.001
**MSC**	4.37e-04	98.557	0.966	0.238	0.137	0.033	0.020	0.012	0.009	0.005	0.003
**SNV**	3.28e-02	97.877	1.534	0.245	0.208	0.041	0.034	0.018	0.010	0.006	0.004
**1D2.15**[d]	3.24e-06	97.702	1.390	0.542	0.150	0.075	0.036	0.034	0.020	0.015	0.009

[a] The total data variance (sum of squares of the mean-centered data); [b] V_1, V_2,… V_{10} – percent variance captured by individual PCs from one to ten; [c] S15 and S47 – variable-wise smoothing with 15- and 47-point window, respectively; [d] Savitzky-Golay first derivative with 2nd order polynomial and 15-point smoothing window.

The PCA analysis performed here provided multivariate proofs of the observations made by visual data inspection (Fig. 1, 3 and Video S-1). Scatter-related variance affecting all spectral variables contains, in addition to a stochastic intensity variation, an essential correlation to the moisture content. Data variable smoothing with an averaging window moving along the time domain effectively eliminates the noise, at that, preserving this correlation and improving the interpretability of process trajectories in the refined factor space. However, due to their spectral similarity, both effects are eliminated by conventional scatter-correction methods operating in the spectral domain, *e.g.* MSC, SNV, and 1st derivative.

S2.3. PLS regression models of moisture content

S2.3.1. Outlying samples

Outlying samples in PLS regression models built on the raw in-line data were detected for each moisture modeling range individually using Q^2- and T^2-Hotelling statistics. Thus, three samples (numbers 35, 99, 144) were excluded from the full-data model resulting in a starting dataset (D) of 298 samples (Table 1 in the paper). Two of them were the samples with the moisture content above 20%, and they fell out when the range was reduced. The sample 35 was repeatedly detected as outlying in data sets D_{20} and D_{15}. In D_{10} it fell out of the range, but another one

(number 36) was eliminated instead. In total, four samples were rejected in different models. Therefore, general data quality was very high, and although the elimination of individual outliers slightly refined the models, it was not a critical step in terms of practical performance of the prediction models.

S2.3.2. Smoothing degree optimization

Time-wise smoothing degree has a significant effect on the modeling accuracy. To optimize it, PLS regression models were built for the windows including 1 (no averaging) to 101 time points (odd numbers only). The optimization was individually performed in different moisture ranges (Fig. S-5): full (D), below 20% (D_{20}), below 15% (D_{15}), and below 10% (D_{10}). In spite of some differences of the global window width (WW), optima observed in the validation *RMSE* and R^2 dependencies (Fig. S-5a and b, respectively), which also depend on the moisture modeling range, there are two important smoothing degrees reflecting the most common trends.

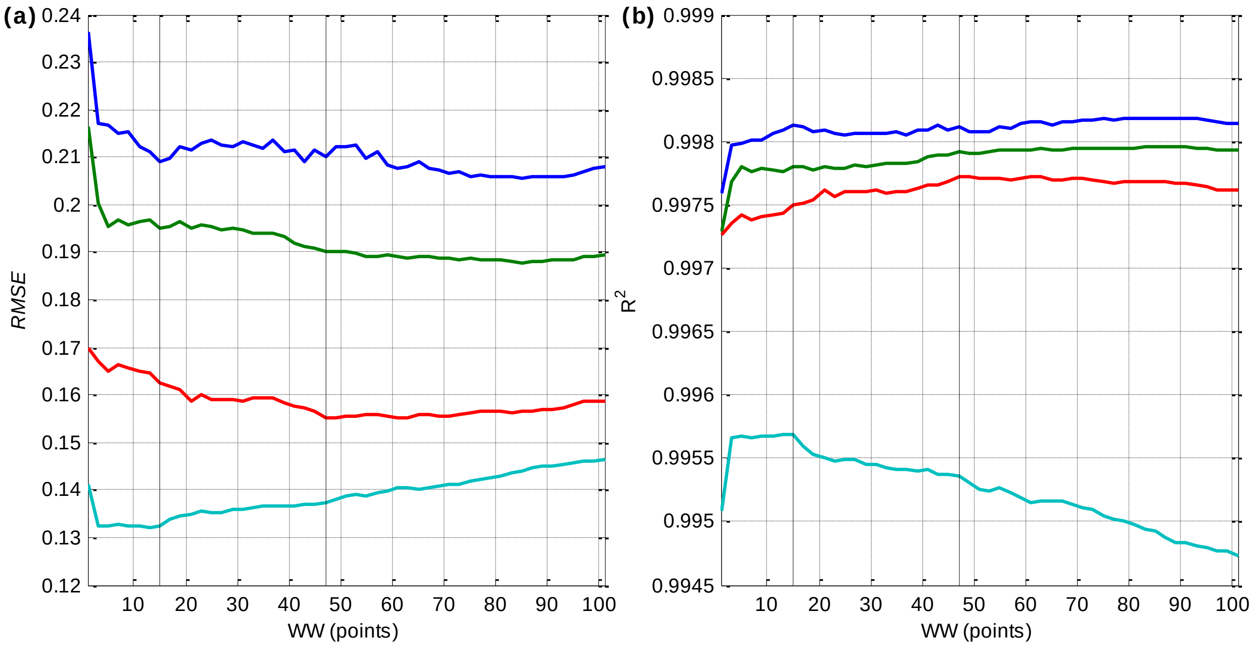

Fig. S-5. Dependences of (a) *RMSE* of LOO CV and (b) R^2 on the smoothing window width (WW) in different moisture content ranges: full (blue), below 20% (green), below 15% (red), and below 10% (light blue); vertical lines indicate important WW values of 15 and 47 points.

The first most essential improvement, compared to the raw data, is already achieved at three to five averaging time points, as corresponds to the process time interval of 15-20 s. Further increase of the averaging degree slowly improves the performance, giving a local R^2 maximum around WW=15. Although the optima of averaging degree are individual for each data set, WW=15 always gives an essential improvement at minimal data transformation, and thus, can

be adopted as a universal preprocessing method in this study. The respective time interval of 70 s approximately corresponds to the full circulation time of the process material in this type of dryer, Thus, each portion of the granulate has a good chance of being exposed to the spectroscopic measurement presented by the average spectrum. After that point the dependences in Fig. S-5 reach different global optima in different moisture content regions, typically, with a very modest gain in model performances compared to WW=15. An exception is D_{10}, where the model validation error with seven LVs keeps falling afterwards. The window of 47 points exhibits the most pronounced global R^2 maximum for D_{15} dataset giving low prediction errors in other datasets, as well. Therefore, smoothing with averaging windows of 15 (S15) and 47 (S47) points have been chosen as benchmarks for the model comparison.

S2.3.3. Deducing the optimal number of LVs

Explained **y**-variances in D_{15} models (Table S-4) reach saturation around 99.8% with approximately seven LVs. Although the **X**-variance exhibits a steeper raise, with the main spectral variance (over 99%) in the first two LVs, it also reaches a plateau after the seventh LV. This is consistent with the former conclusion that seven factors are necessary to describe relevant **X**-variance in the PCA model of bulk process spectra (section S2.2.2).

Table S-4. Cumulative **X**- and **y**-variance captured by different numbers of LVs in PLS regression models.

nLV[a]	D_{15} (raw) [b]		D_{15} (S15)		D_{15} (S47)		D_{15} (MSC)	
	Vx[c]	*Vy*	*Vx*	*Vy*	*Vx*	*Vy*	*Vx*	*Vy*
**1**	92.814	75.478	96.894	92.135	97.455	93.378	98.064	95.505
**2**	99.561	97.446	99.469	98.063	99.493	98.263	99.075	98.371
**3**	99.858	99.047	99.828	99.424	99.831	99.577	99.625	99.195
**4**	99.941	99.366	99.931	99.543	99.937	99.643	99.732	99.482
**5**	99.976	99.507	99.971	99.622	99.972	99.699	99.872	99.609
**6**	99.984	99.709	99.981	99.751	99.981	99.786	99.914	99.660
**7**	99.989	99.781	99.987	99.798	99.986	99.817	99.944	99.710
**8**	99.990	99.805	99.992	99.813	99.992	99.827	99.949	99.800
**9**	99.994	99.817	99.994	99.833	99.994	99.843	99.956	99.825
**10**	99.996	99.842	99.995	99.860	99.995	99.864	99.959	99.850
**Total[d]**	**1.62e-3**	**1.05e+1**	**1.15e-3**	**1.05e+1**	**1.12e-3**	**1.05e+1**	**1.62e-4**	**1.05e+1**

[a] The number of LV in a PLS regression model; [b] D_{15} – data subset of samples with moisture content <15% (Table 1 in the main paper) with applied preprocessing given in brackets (see footnote to Table S-3); [c] cumulative per cent **X**- (*Vx*) and **y**- (*Vy*) variances in the calibration dataset; [d] total **X**- and **y**-variances in the calibration dataset.

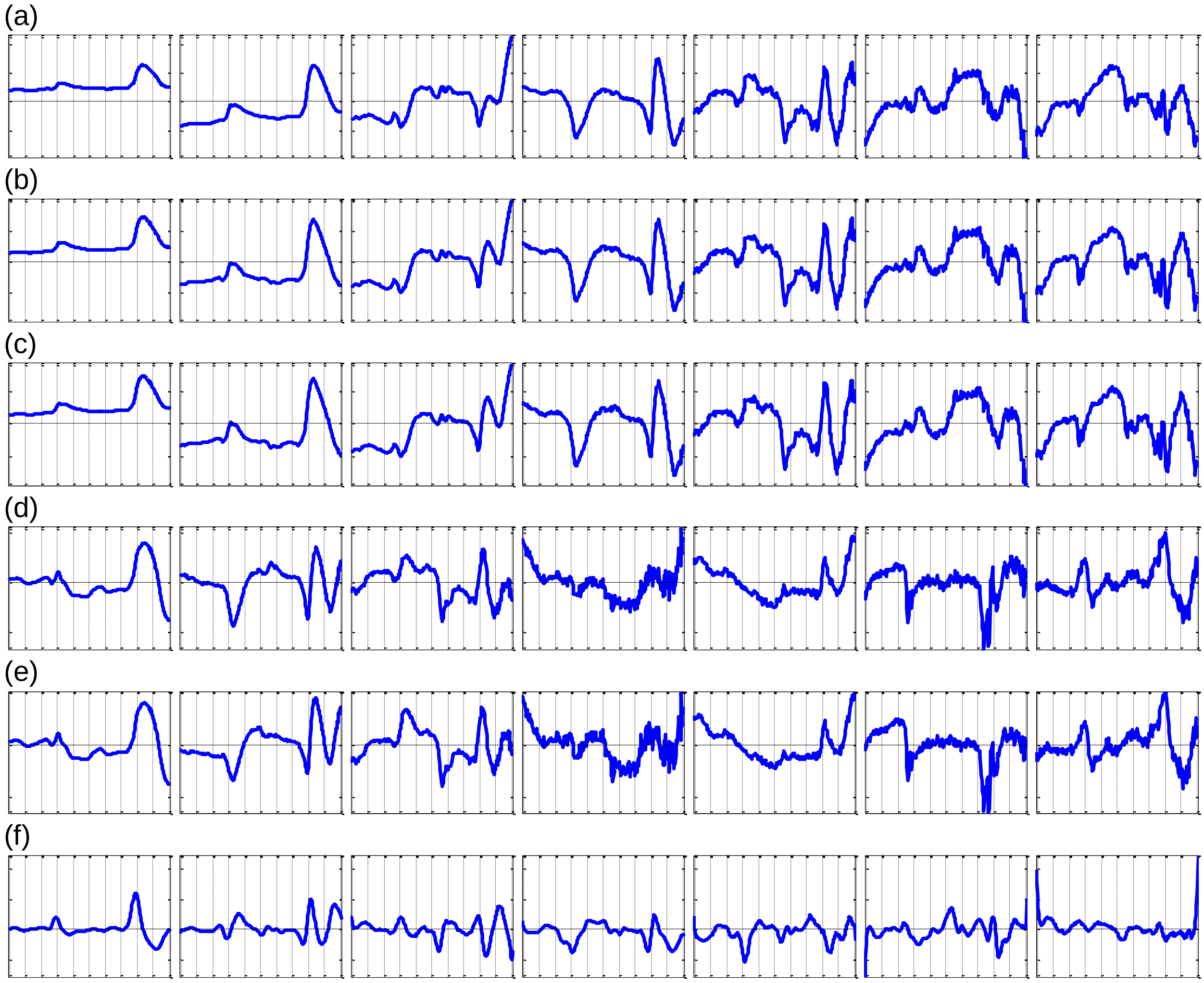

Fig. S-6. PLS regression loading linear plots versus wavelength (**p**$_1$-**p**$_7$, left to right) for different data preprocessing methods: (a) none; (b) and (c) variable smoothing with a 15- and a 47- point window: S15 and S47, respectively; (d) MSC; (e) SNV; and (f) first derivative by Savitzky-Golay. All loadings of the same model (**p**$_1$-**p**$_7$) have the same y-scales.

For the raw and averaged spectral data (*i.e.* retaining their measurement units) significances of individual PLS LVs have been additionally proved using the approach suggested in section S2.2.2. It has been shown that **X**-variable standard deviations corresponding to individual PLS factors keep values above the spectrometer noise (0.0005 absorbance units) up to the seventh LV, at least. Similar analysis performed on the **y**-vector also justified the significance of the first seven PLS factors. For instance, standard deviation of the moisture content captured by LV$_7$ in D is 0.084; this is comparable to experimentally determined reproducibility error of the LOD analysis (Table S-1 in section S1.2). Therefore, although percent values of **X**- and **y**-variances captured by minor PLS factors (specifically by LV$_7$) in presented models may seem extremely low (Table S-4), they are still in agreement with the measurement precisions of respective analytical techniques. This traceability to the source data, enabling a direct significance

justification of individual LVs in a multivariate model, is an additional advantage of avoidance of the conventional scatter-correction prior to the multivariate modeling. The differences in validation *RMSE* and explained **y**-variances for six and seven LVs are getting smaller with the averaging degree (Fig. 4b and Table S-4). Considering these results, seven LVs were found to be the optimal model complexity for different moisture ranges and data preprocessing methods. This number is also reasonable considering process physical and chemical variability as well as complex nature, and hence possible non-linearity of spectral responses. Meaningful PLS loadings in Fig. S-6 provide an additional argument for a PLS regression models with seven LVs. Note that PLS (Fig. S-6) and augmented PCA loadings (Fig. S-4) are almost identical; therefore, both multivariate modeling spaces are essentially the same and conclusions about the model complexity for PCA made in section 2.2.2 are basically valid in the PLS model case.

Close examination of the model complexity issue performed here was necessary, because the majority of previously published PLS regression models of the moisture content in a fluidized bed drying monitored by the in-line NIR spectroscopy typically used 2-5 LVs [G.X. Zhou, Z. Ge, J. Dorwart, B. Izzo, J. Kukura, G. Bicker, J. Wyvratt, Determination and differentiation of surface and bound water in drying substances by near infrared spectroscopy, Journal of Pharmaceutical Sciences 92 (2003) 1058–1065; R.L. Green, G. Thurau, N.C. Pixley, A. Mateos, R.A. Reed, J.P. Higgins, In-line monitoring of moisture content in fluid bed dryers using near-IR spectroscopy with consideration of sampling effects on method accuracy, Analytical Chemistry 77 (2005) 4515–4522; J. Mantanus, E. Ziémons, P. Lebrun, E. Rozet, R. Klinkenberg, B. Streel, B. Evrard, Ph. Hubert, Moisture content determination of pharmaceutical pellets by near infrared spectroscopy: Method development and validation, Analytica Chimica Acta 642 (2009) 186–192; M. Alcala, M. Blanco, M. Bautista, J. M. González, On-Line monitoring of a granulation process by NIR spectroscopy, Journal of Pharmaceutical Sciences, 99 (2010) 336–345; A. Burggraeve, A. F. T. Silva, T. van den Kerkhof, M. Hellings, C. Vervaet, J. P. Remon, Y. van der Heyden, T. De Beer, Development of a fluid bed granulation process control strategy based on real-time process and product measurements, Talanta 100 (2012) 293–302]. Lower model complexity, however, must have been a consequence of insufficient volume or representativeness of the calibration data set. Indeed, limiting our data to subsets, *e.g.* with the same API, reduces the model optimal dimensionality. Thus, for single batches or for a combination of similar process runs it falls down to 2-4 LVs (Table S-5). Model simplification in partial datasets is conditioned, on one hand, by the reduced process diversity, and hence spectral variability. On the other hand, the number of samples may be deficient for building a full-featured model of higher dimensionality. In the latter case, scatter-correction methods strengthening the first PLS factors become advantageous.

Table S-5. PLS modeling statistics for reduced datasets (nLV, *RMSE* and R^2 are estimated by LOO CV).

Dataset	nS[a]	Raw data			MSC		
		nLV	*RMSE*	R^2	nLV	*RMSE*	R^2
B03	20	4	0.169	0.9992	4	0.336	0.9968
B22	24	3	0.172	0.9987	2	0.287	0.9965
B23	26	3	0.154	0.9979	2	0.181	0.9967
B24	24	3	0.141	0.9981	2	0.225	0.9953
B01-B08[b]	80	4	0.380	0.9961	2	0.568	0.9913
B09-B16[c]	80	4	0.208	0.9980	3	0.287	0.9961
B17-B24[d]	123	3	0.171	0.9983	5	0.210	0.9975

[a] The number of samples in the subset; [b] 0.1 mg API assay; [c] 1.0 mg API assay; and [d] 10 mg API assay. For batch description see Table S-1.

S2.3.4. Predicted process trajectories

Predicted time dependencies of the moisture content for B12 are presented in Fig. S-7. Comparing them, it is possible to make additional conclusions about the efficiency of different preprocessing methods for the process monitoring performance. Time-domain smoothing is much more efficient to reduce the noise spoiling the trajectories at the process beginning, where the moisture content is above 15%. At this process stage the material is sticky and may foul the probe. First derivative is the least effective method to for the noise reduction. SNV and to a lesser extent MSC tend to exaggerate the corrective effect of the probe cleaning on predicted moisture content in the surrounding medium, leading to the appearance of positive artifacts. (It should be noted that this is an extreme case; in general, the spectral variance related to clean and fouled-up probe states is successfully handled by the seven-LV model itself, see Fig. S-8.) This susceptibility of the predicted process curve to various interferences is the main reason of worse modeling accuracy during the “wet” process stage. Model accuracies below 15% are higher for all preprocessing methods; and their differences of performance can hardly be discovered by visual inspection of the process curves. Switching between the process technological phases does not have any noticeable effect on the prediction.

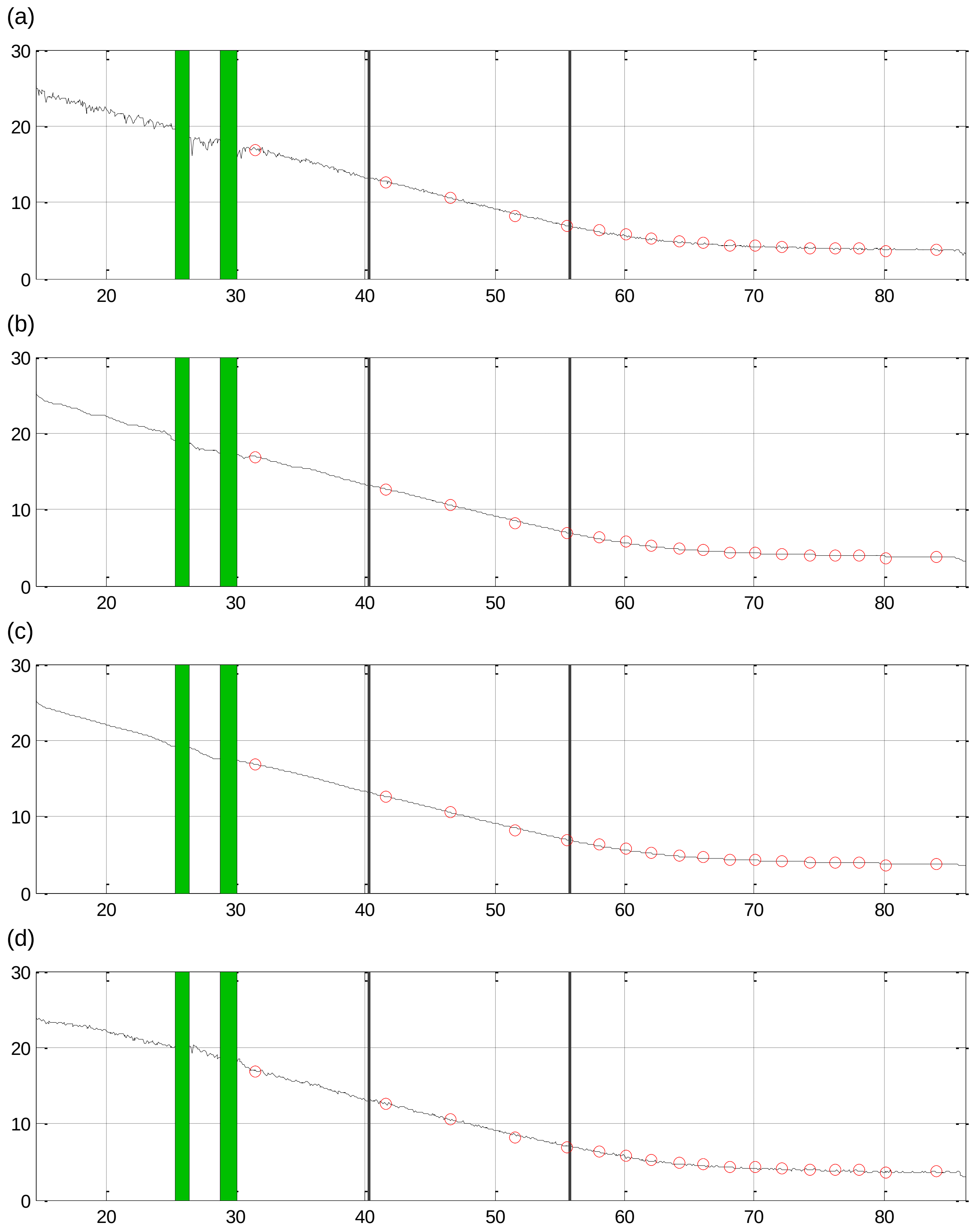

(a)
30
20
10
0
20
30
40
50
60
70
80
(b)
30
20
10
0
20
30
40
50
60
70
80
(c)
30
20
10
0
20
30
40
50
60
70
80
(d)
30
20
10
0
20
30
40
50
60
70
80

(e)

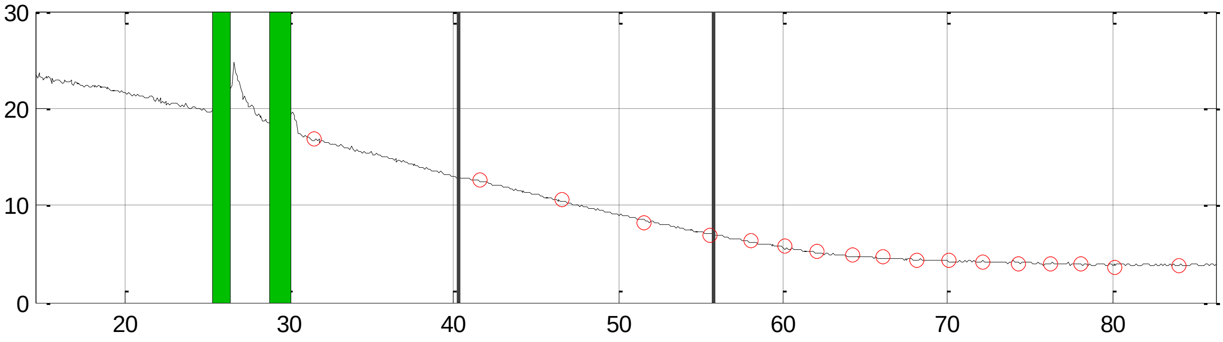

(f)

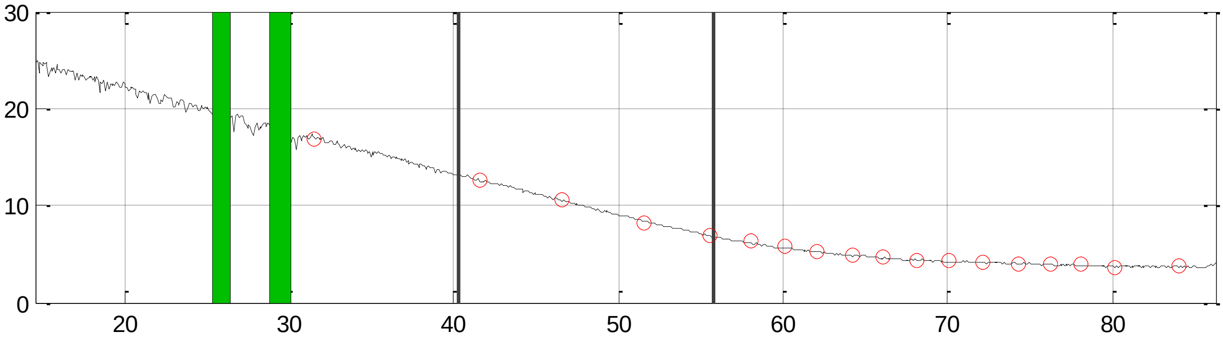

Fig. S-7. Predicted moisture content versus process time (min) in B12 using full-dataset PLS regression model (D in Table 1) and different preprocessing methods: (a) none, (b) and (c) - time-domain smoothing with a 15- and 47-point window: S15 and S47, respectively; (d) MSC; (e) SNV; and (f) first derivative by Savitzky-Golay. Red circles indicate reference values of LOD sample analyses; green intervals designate the probe cleaning cycles, and vertical black lines – changes of the process technological phases.

Overlaid view of process trajectories obtained using three different preprocessing techniques (no preprocessing, S15 and MSC) for all 25 batches is presented in Fig. S-8. Direct comparison of the methods makes it clear that MSC is the least advantageous preprocessing, particularly inaccurate at the process beginning stage.

[see “Image 2.pdf” file]

Fig. S-8. Predicted moisture content versus process time (min) in all 25 batches (the batch numbers are indicated at the bottom left corner of each plot) using full-dataset PLS regression model (D in Table 1) and different preprocessing methods. The curve color designates the preprocessing method used: black – none, red – variable smoothing with a 15-point averaging window (S15); blue – MSC; all the models use 7 LVs. Red circles indicate reference values of LOD sample analyses; green intervals designate the probe cleaning cycles, and vertical black lines – changes of the process technological phases.

S2.3.5. Off-line FT-NIR analysis of selected samples

A subset of 101 selected process samples (Table S-1) were analyzed off-line using a lab FT-NIR spectrophotometer (section S1.5) and PLS regression models of the moisture content were built. The respective set of in-line spectra of the same samples were used for comparison. The results are presented in Table S-6.

Application of an integrating sphere (IS) prevents the loss of light that is scattered by the sample, thus minimizing random effects caused by the granulate surface roughness and density fluctuations on the spectral intensity. At the same time, the total path length travelled by the light in a sample, and hence its absorption by the granulate constituents, stays dependent on the moisture content resulting in a fall of spectrum background in the whole studied range (Fig. S-9). Similar behavior of off-line NIR spectra was previously studied using pharmaceutical excipient (microcrystalline cellulose) and inorganic glass beads (“ballotini”) with a known size distribution [J. Rantanen, E. Räsänen, J. Tenhunen, M. Känsäkoski, J.-P. Mannermaa, J. Yliruusi, In-line moisture measurement during granulation with a four-wavelength near infrared sensor: an evaluation of particle size and binder effects, European Journal of Pharmaceutics and Biopharmaceutics 50 (2000) 271–276] and explained by the difference of refractive indices on the crystal-air and crystal-water interfaces. Correlation dependence of the spectral intensities on the water content at all possible wavelengths (Fig. S-10) exhibits generally high relationship of the spectral variables outside the main water absorption regions, as it was observed for the in-line data (Fig. 2b in the paper). Exaggeration of the minor spectral features in the correlation curve confirms its sensitivity to the light absorption by different mixture components due to their indirect correlation with the moisture content that affects the measurement path length. Higher contrast of the curve corresponding to off-line data compared to the respective dependence built for in-line spectra (black and grey curves in Fig. S-10) is accounted for by higher spectroscopic resolution of the FT-NIR method. The observed correlation is generally higher in off-line spectra, but data smoothing along the time scale using a 47-point averaging window (red dashed curve in Fig. S-10) makes the correlations as high as (and on some wavelength regions even higher as) in the off-line data. Higher correlations can be explained by a smaller (about 2 g) off-line sample volume compared to the virtual sample exposed to the in-line spectral measurement in the reaction bowl that provides practically infinite light penetration depth. The physical integrating effect of IS on the spectral measurements that eliminates the random scatter while preserving useful moisture-related information is similar to mathematical averaging applied to in-line data.

Table S-6. PLS regression models for in- and off-line NIR spectra of selected process samples.

Dataset	nS[a]	PP[b]	nLV	Calibration		LOO CV	
				RMSE	R^2	*RMSE*	R^2
**D_{off}**[c]	**101**	**none**	6	0.128	0.9987	0.150	0.9982
		**MSC**	7	0.116	0.9989	0.157	0.9981
		**SNV**	7	0.120	0.9989	0.156	0.9981
		**1D2.15**	7	0.094	0.9993	0.127	0.9987
**D_{off15}**[d]	**96**	**none**	6	0.096	0.9989	0.108	0.9986
		**MSC**	7	0.089	0.9990	0.108	0.9986
		**SNV**	7	0.092	0.9990	0.111	0.9985
		**1D2.15**	5	0.074	0.9993	0.107	0.9986
**D_{in15}**[e]	**96**	**none**	6	0.101	0.9988	0.113	0.9985
		**S15**	6	0.095	0.9989	0.108	0.9986
		**S47**	6	**0.088**	**0.9991**	**0.099**	**0.9988**
		**MSC**	7	0.084	0.9991	0.119	0.9983
		**SNV**	7	0.091	0.9990	0.127	0.9980
		**1D2.15**	6	0.098	0.9989	0.113	0.9985

[a] The number of samples in the dataset; [b] preprocessing method (see footnote to Table S-3); [b] full dataset for off-line analysis; [c] off-line analysis dataset with moisture content <15%; [d] in-line analysis dataset with moisture content <15%, *i.e.* the same samples (and their reference moisture values) as in D_{off15} and corresponding in-line spectra.

Limiting the modeling to 96 (out of 101) samples where the moisture content was below 15% resulted in a significant improvement of prediction accuracy. *RMSE* of LOO CV fell from 0.150 in D to 0.108 in D_{off15} for non-preprocessed data. Therefore, direct comparison of PLS models built for off- and in-line spectral data was made on the same reduced sample set (in Table S-6 they are designated as D_{off15} and D_{in15}, respectively). Here, the FT-NIR laboratory analysis offering much higher spectral resolution and optimized measurement conditions can be considered as a “gold standard”. Therefore, its direct comparison with an in-line analysis presents a methodological value and helps to to evaluate the effects of analysis transfer into a running process environment.

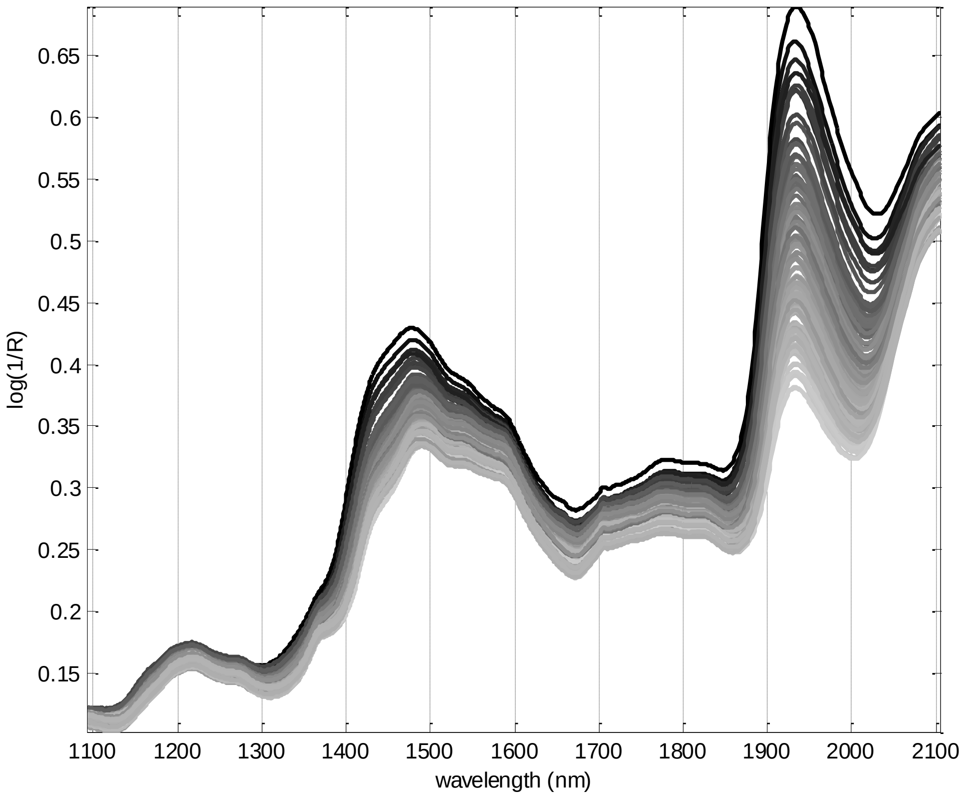

Fig. S-9. Off-line NIR spectra of 101 selected process samples; moisture content is coded by the greyscale colors (black for maximal LOD value in the samples).

As one could expected, the performance of a PLS model for the raw in-line data D_{in15} is lower than in the case of non-processed laboratory FT-NIR spectra. However, a moderate data smoothing (S15) already brings it to the same level as for D_{off15}, while stronger smoothing (S47) leads to a noticeable advantage of the in-line model. This result agrees with a generally higher, compared to the off-line spectra, wide-range correlation of smoothed spectral variables in the process data (Fig. S-10). Higher resolution of FT-NIR spectra does not play any essential role in the quantitative modeling. In both in- and off-line cases the models were built for six LVs. Some model simplification compared to the full-data in-line calibration is explained by a reduction of the sample set size, and thus, its representativeness, as previously discussed in section S2.3.3.

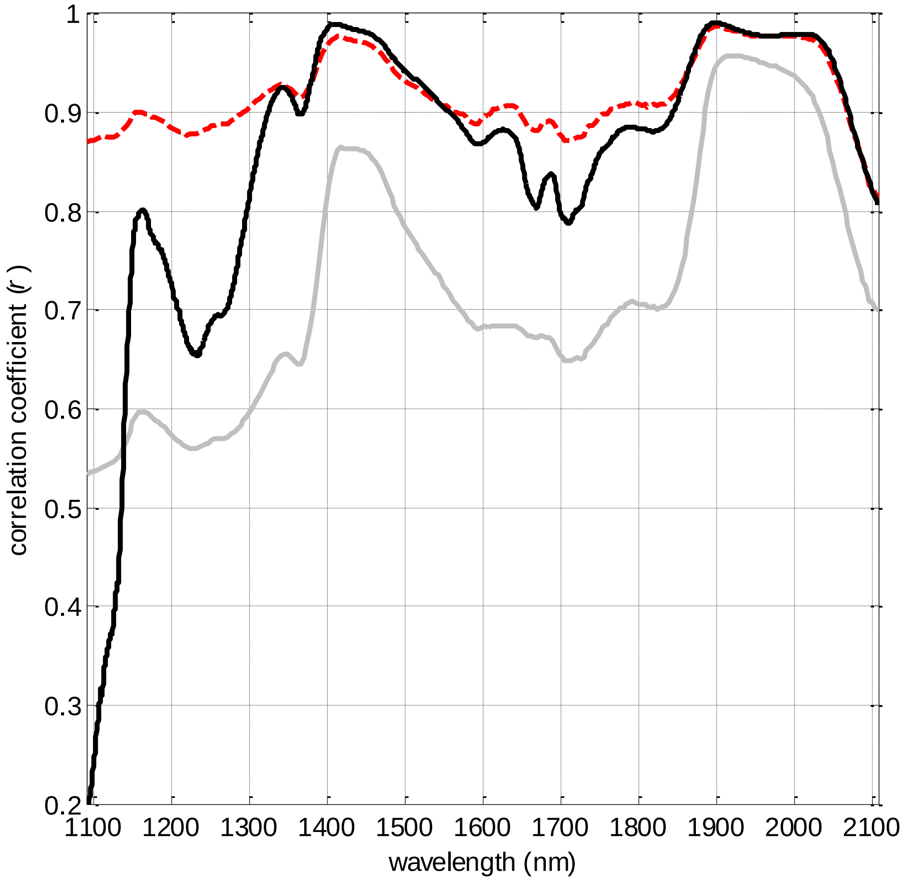

Fig. S-10. Correlation coefficients (*r*) between the moisture content (LOD) and spectral intensities at all individual wavelengths for the set of 101 process samples in: off-line FT-NIR spectra (black solid), in the raw in-line (grey) and in the in-line data smoothed (S47) along the time scale (red dashed).

Scatter correction of the laboratory FT-NIR spectra does not bring any noticeable gain; moreover, PLS model prefaced by MSC or SNV require seven LVs to describe the relevant data variance (Table S-6). Perhaps, IS application makes this corrective preprocessing redundant.

S3. Extended conclusions

Beside its utilitarian value, this study contributes to general understanding of diffuse reflectance NIR spectroscopy as an in-line analytical method for the moisture monitoring in powder processing. The acquired new knowledge that was used to reach a new monitoring accuracy level in the studied process, can also serve as a guidance for making informed decisions, *e.g.* on experimental design, data preprocessing choice and modeling strategy, in similar studies.

In general, this work illustrates a well-known fact that the maximum performance of analysis is reached through an in-depth understanding of the analyzed object (*e.g.* process) and analytical technique. This knowledge can be obtained by thorough exploratory analysis of a designed representative set of experimental data.
