# Supplementary figures and images for: Accuracy Improvement of In-line Near-Infrared Spectroscopic Moisture Monitoring in a Fluidized Bed Drying Process
Source: Front Chem. 2018 Oct 10;6:388. doi: 10.3389/fchem.2018.00388 (PMC6192013; doi:10.3389/fchem.2018.00388)

(a) none

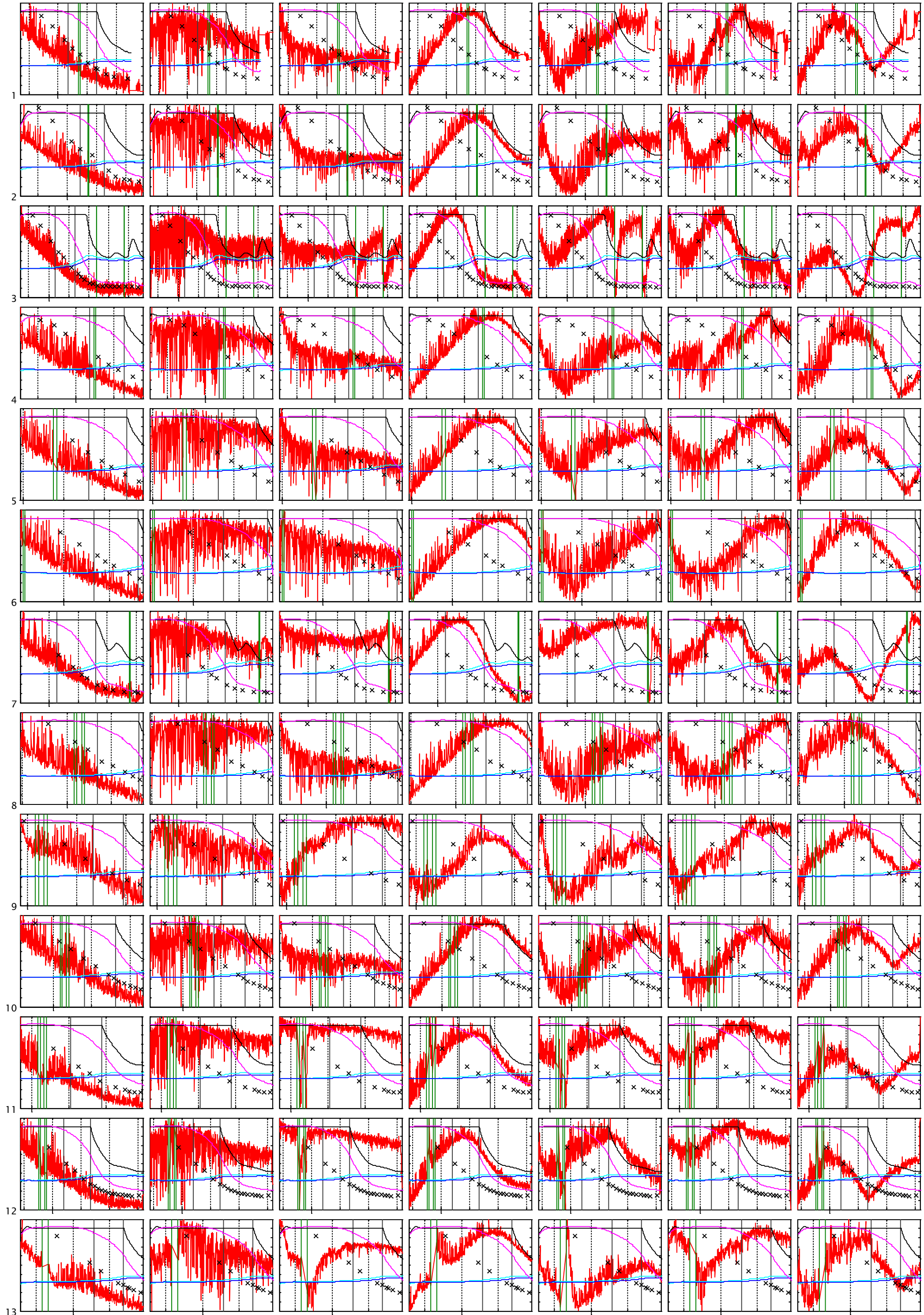

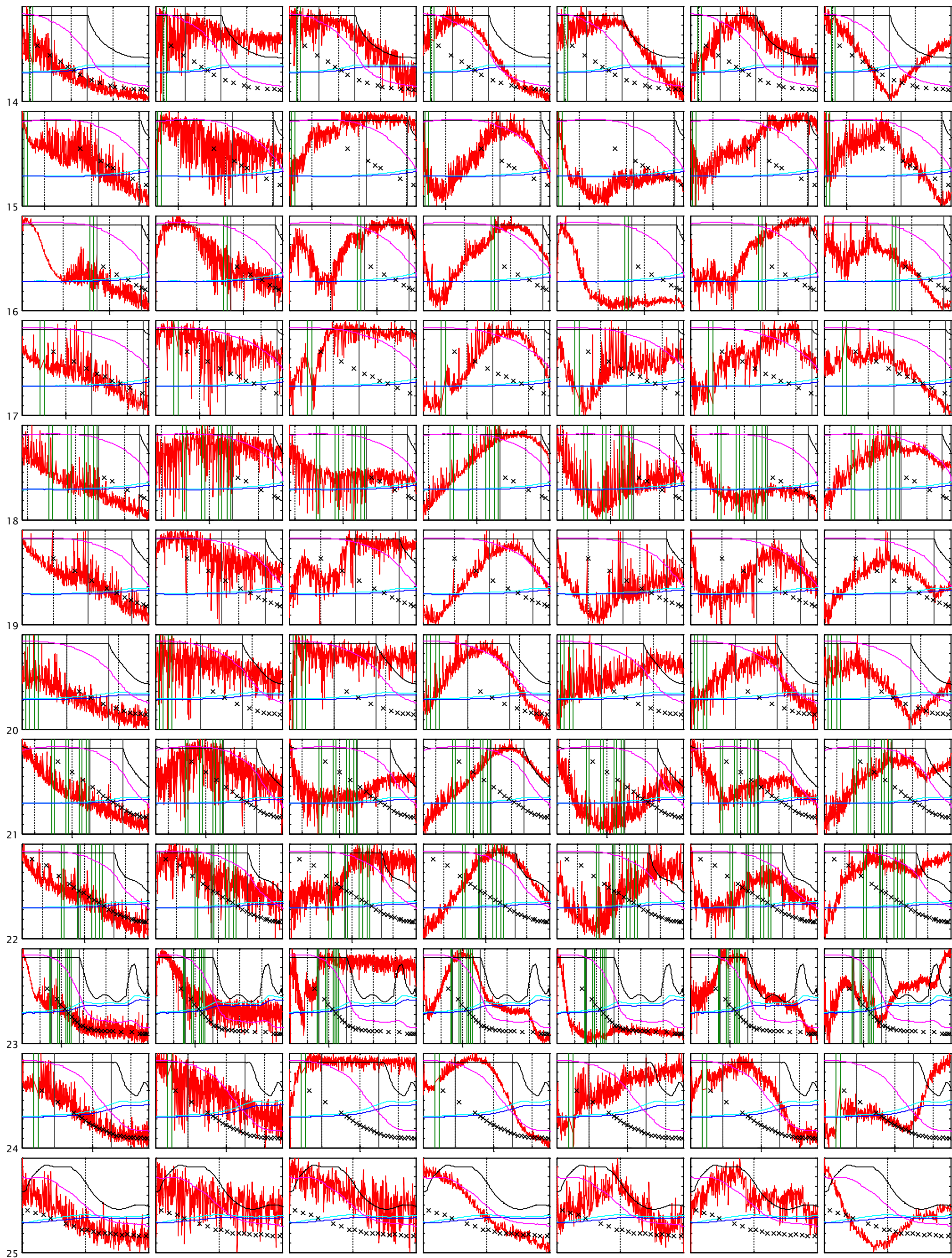

(b) S15

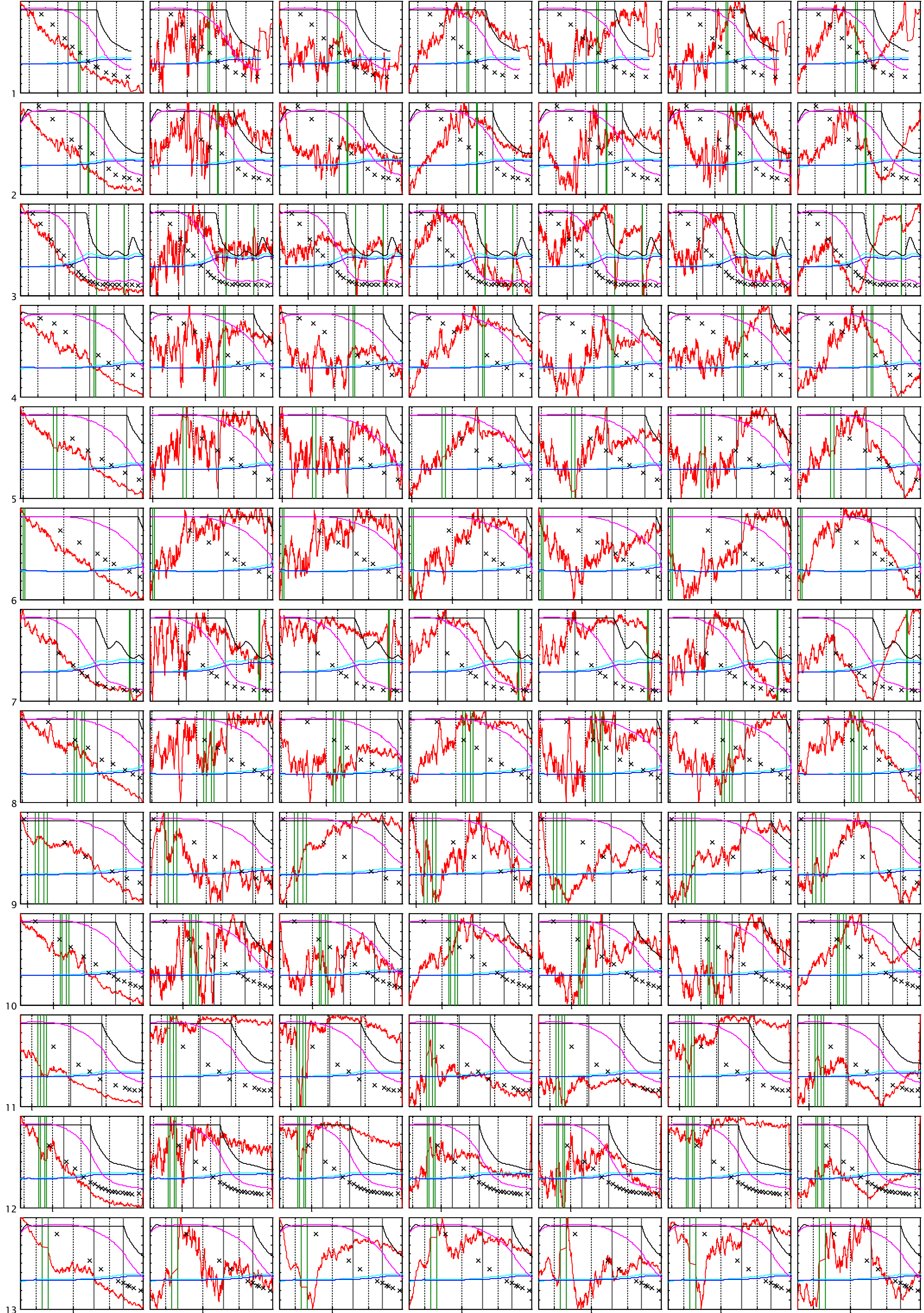

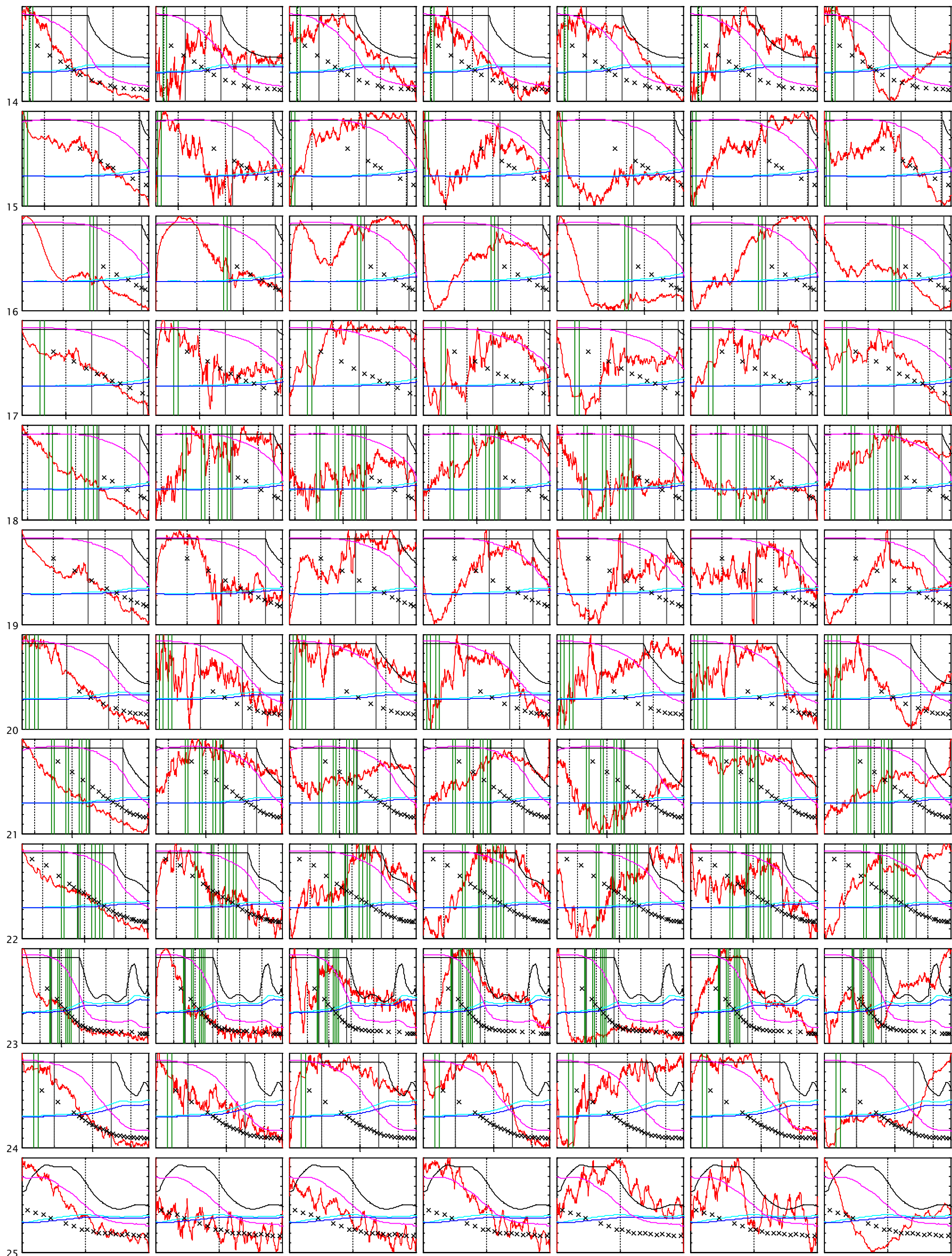

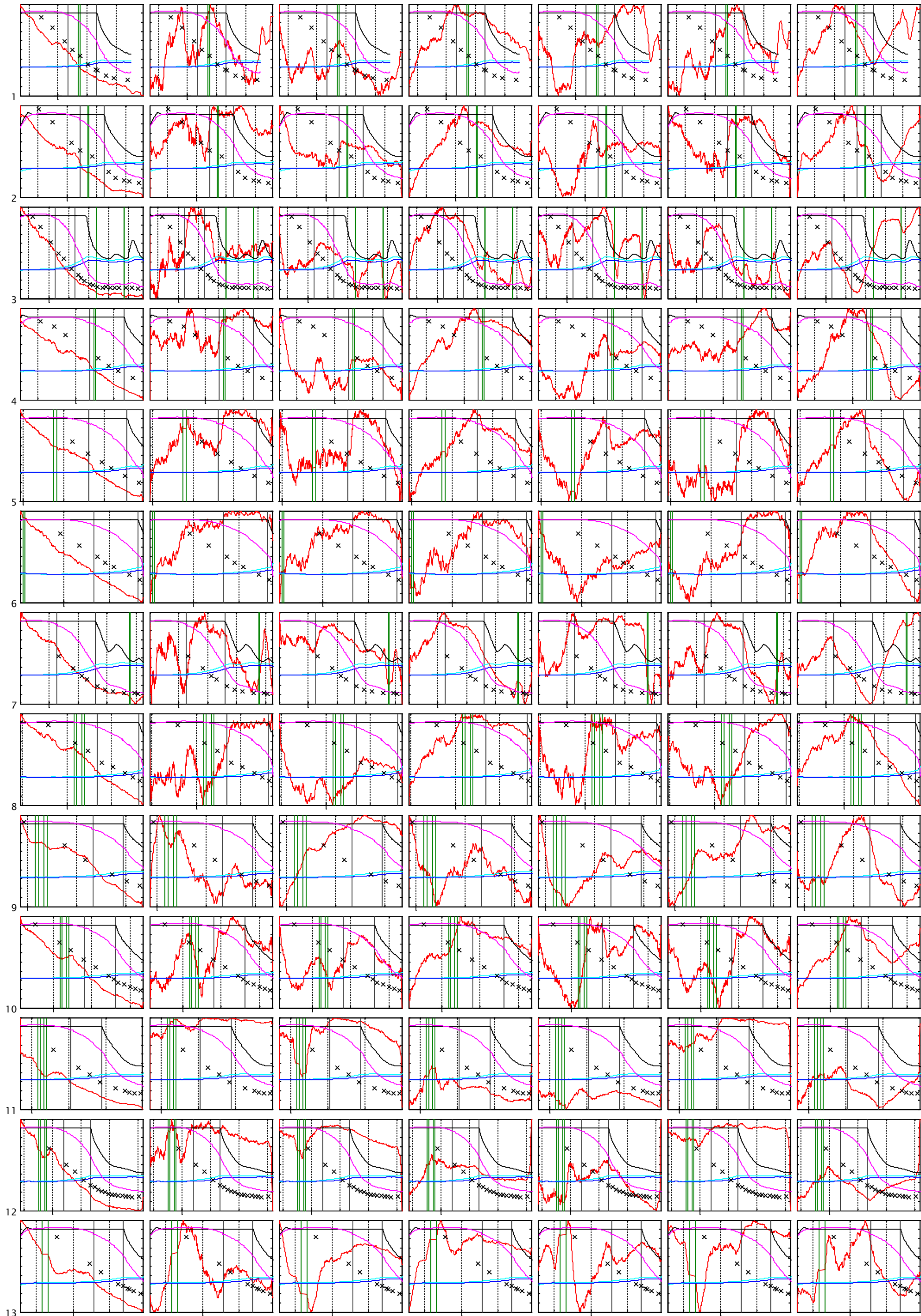

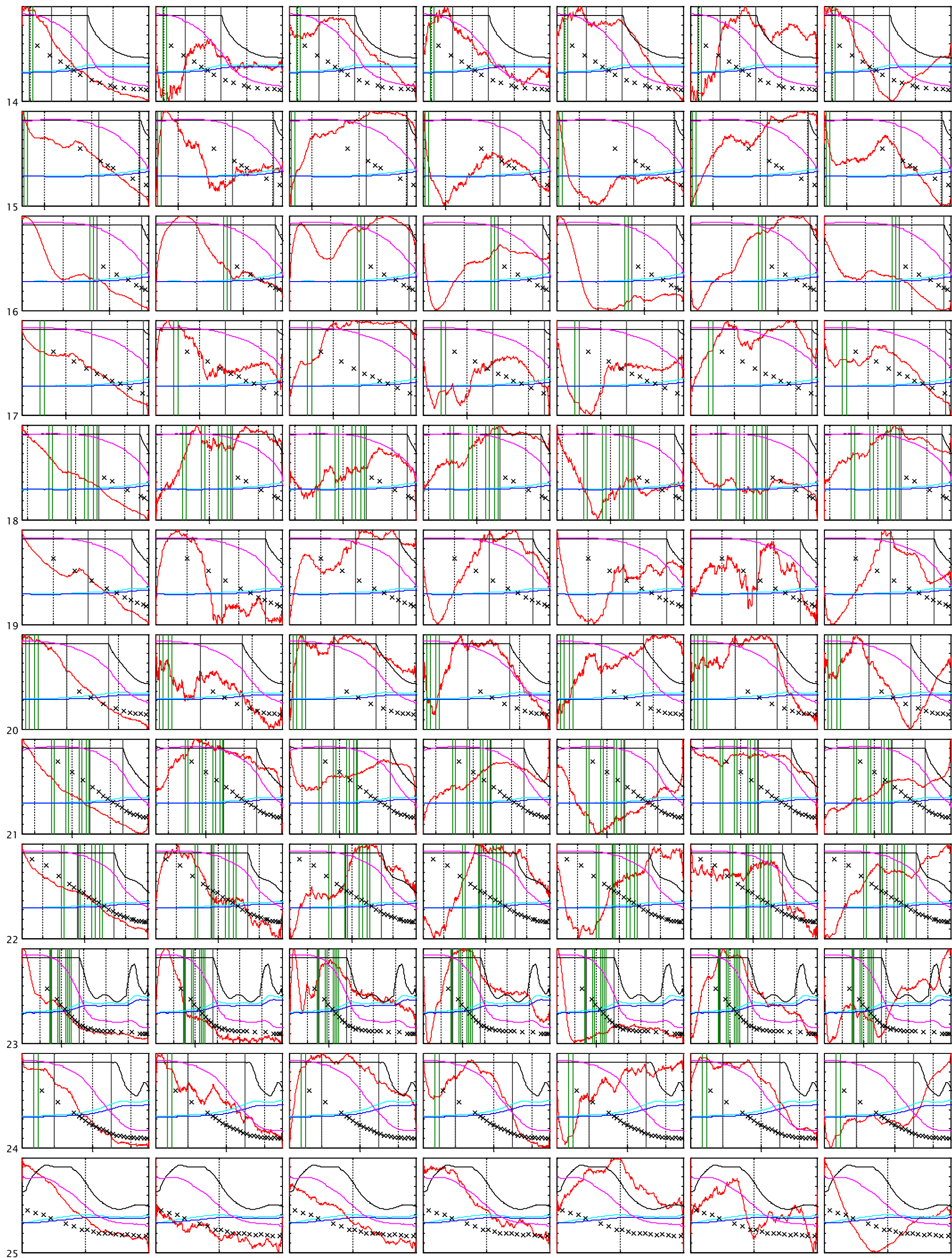

(d) MSC

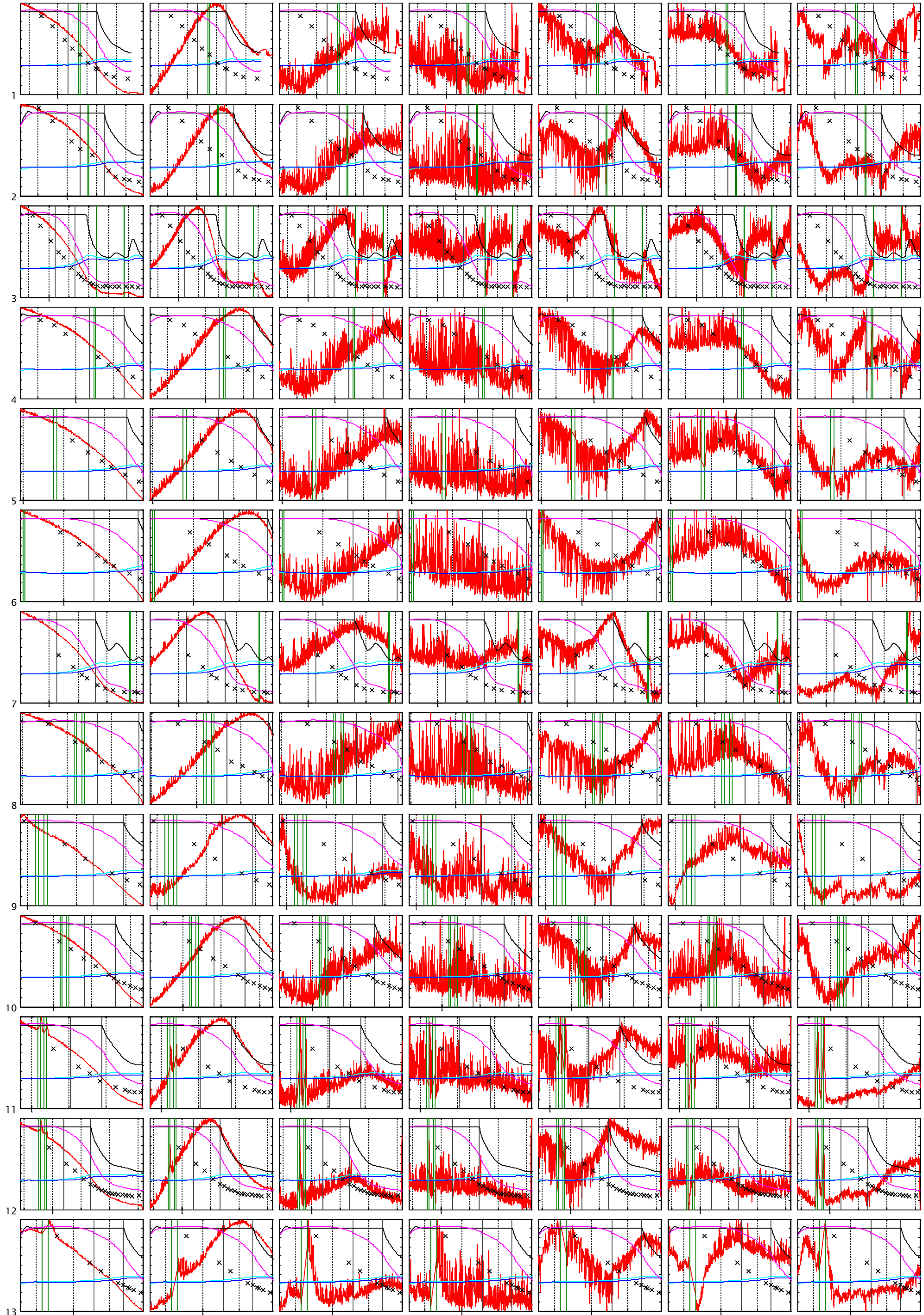

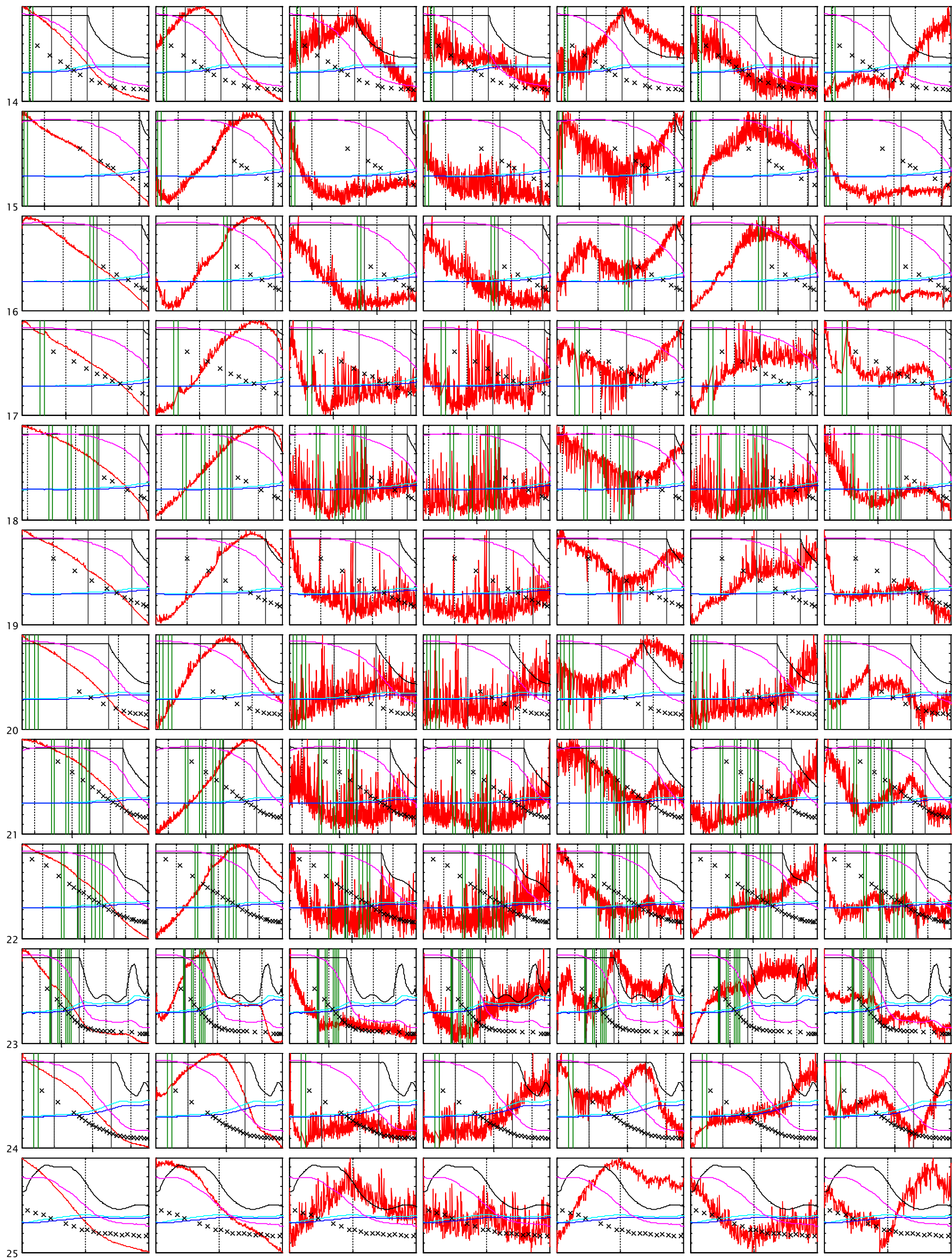

(e) SNV

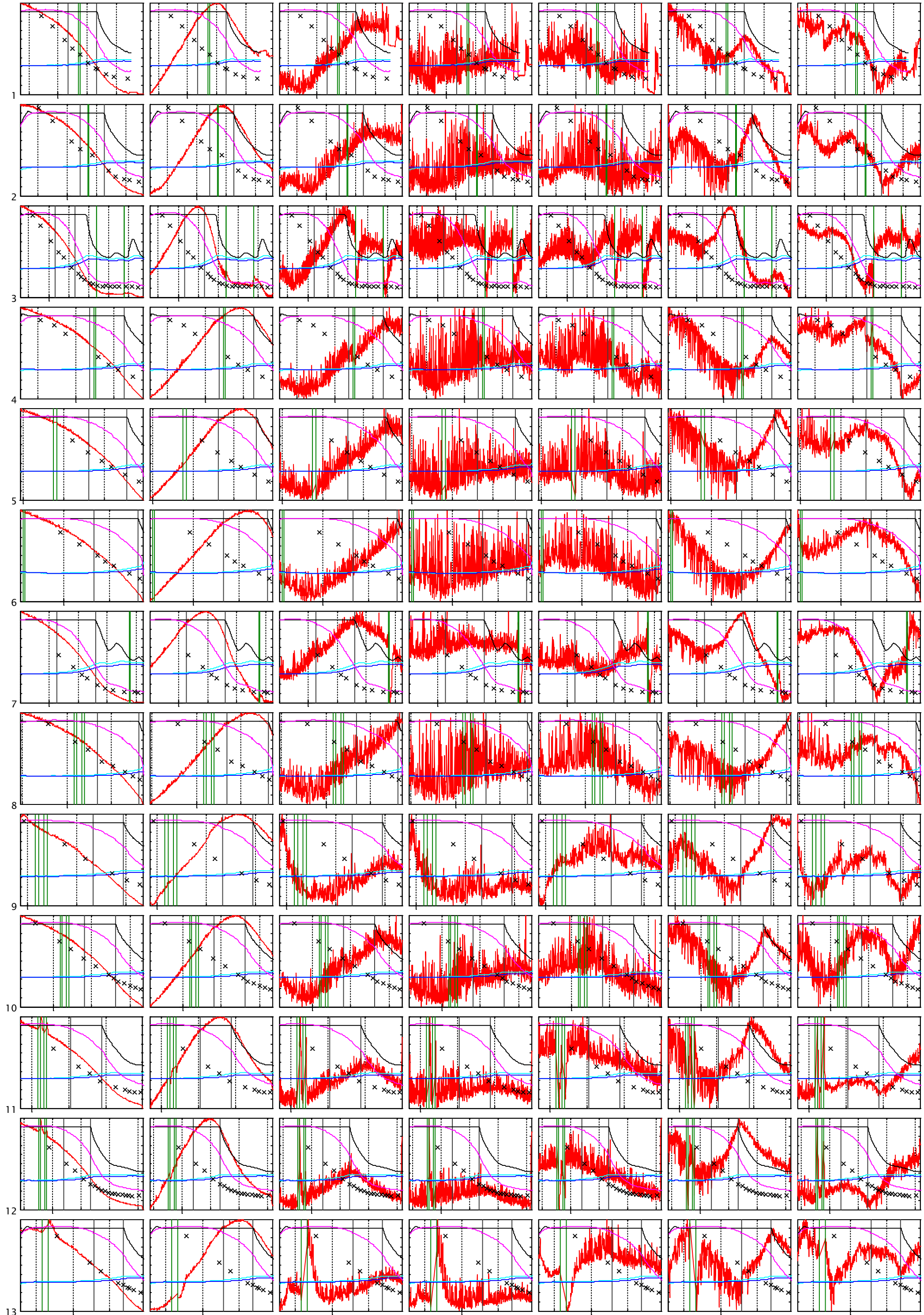

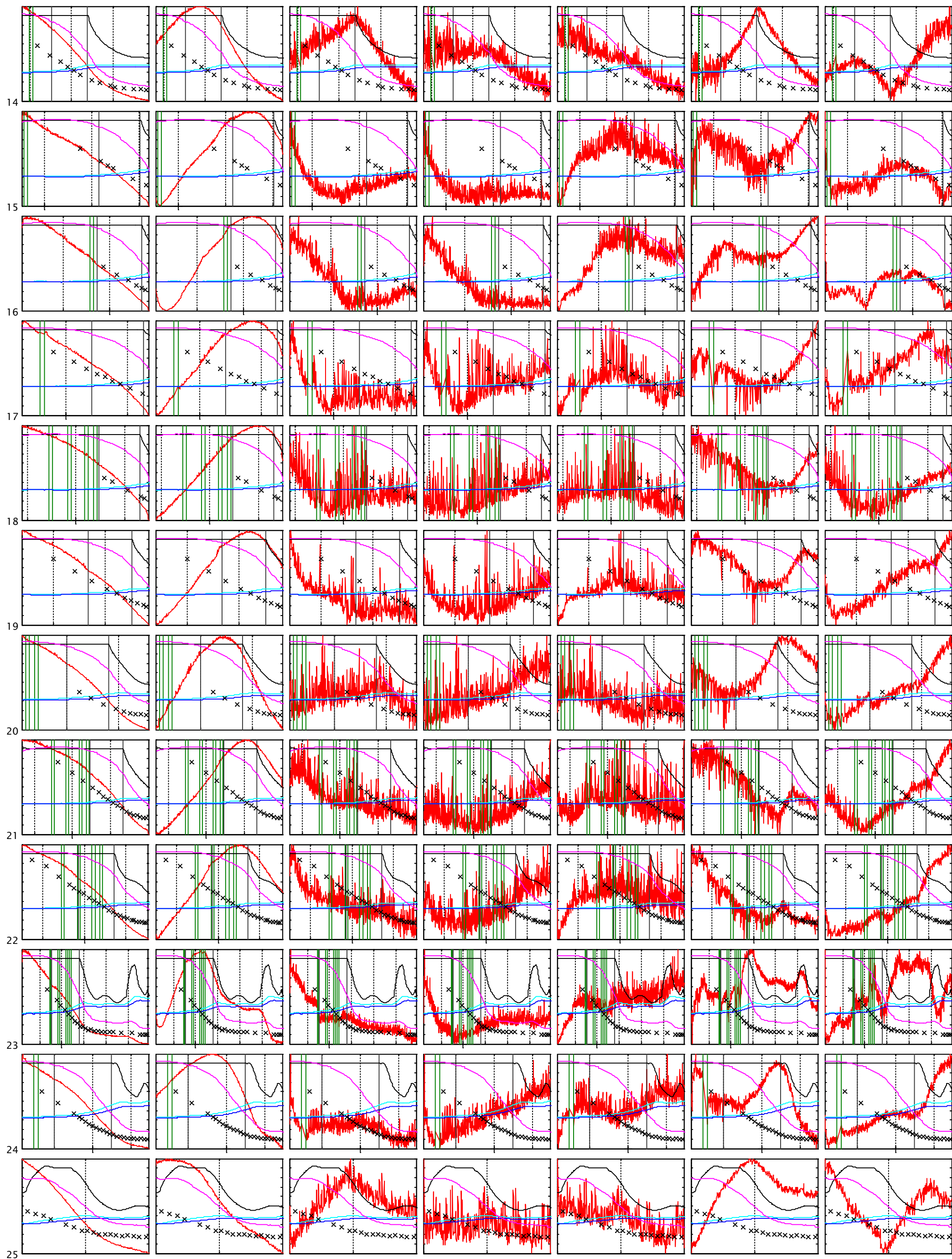

(f) 1D2.15

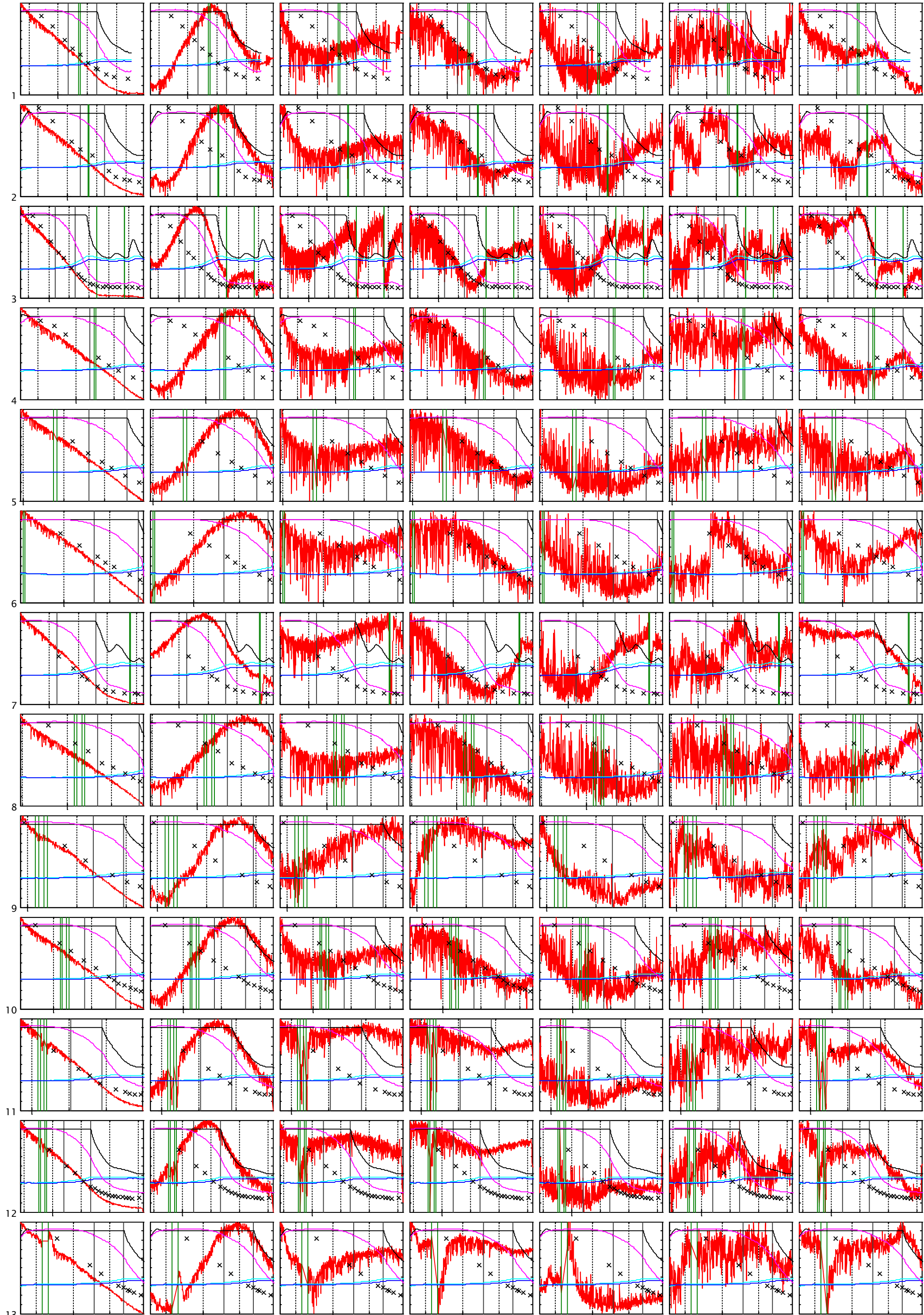

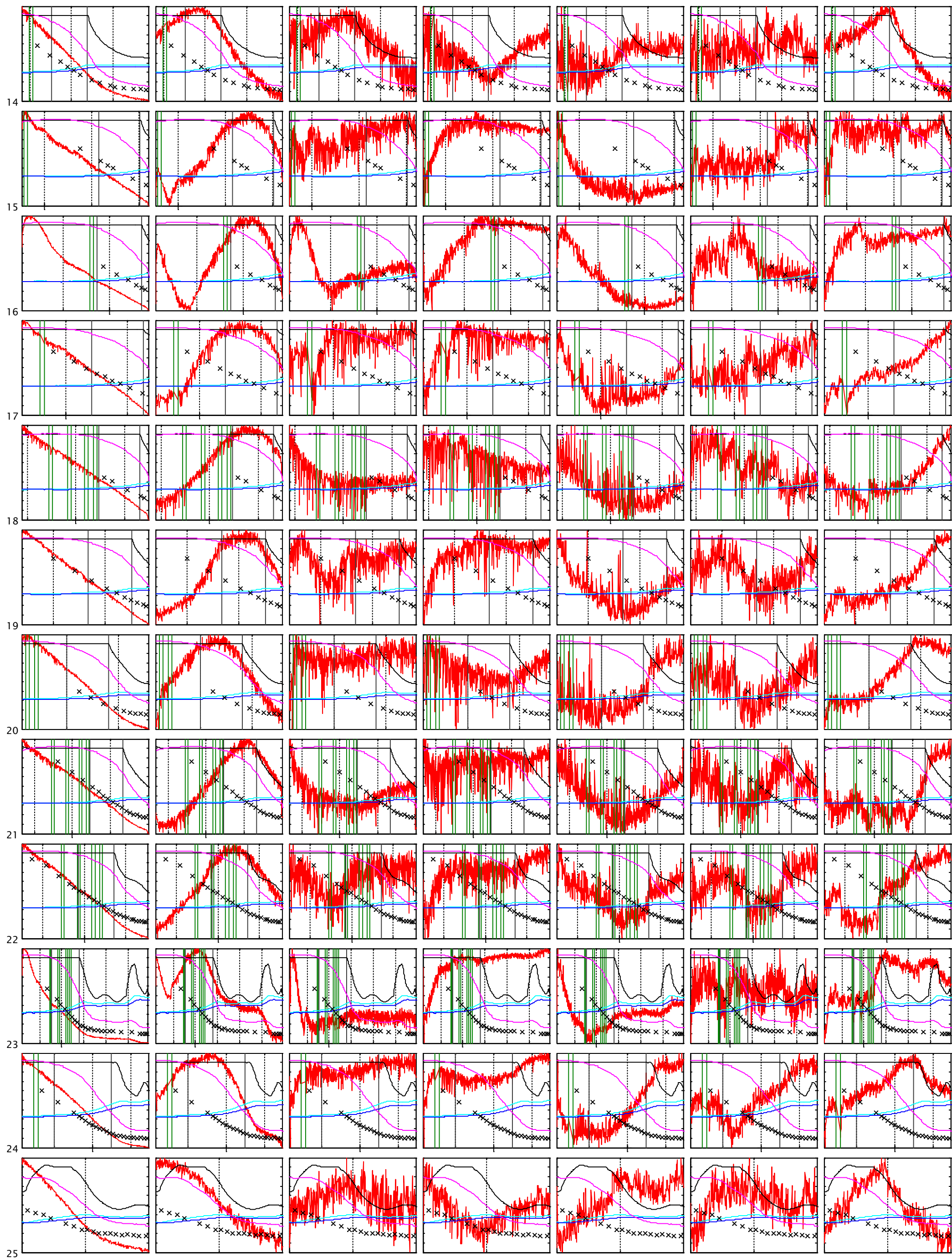

Supplement: Supplementary file 3 [file Image_1.pdf]

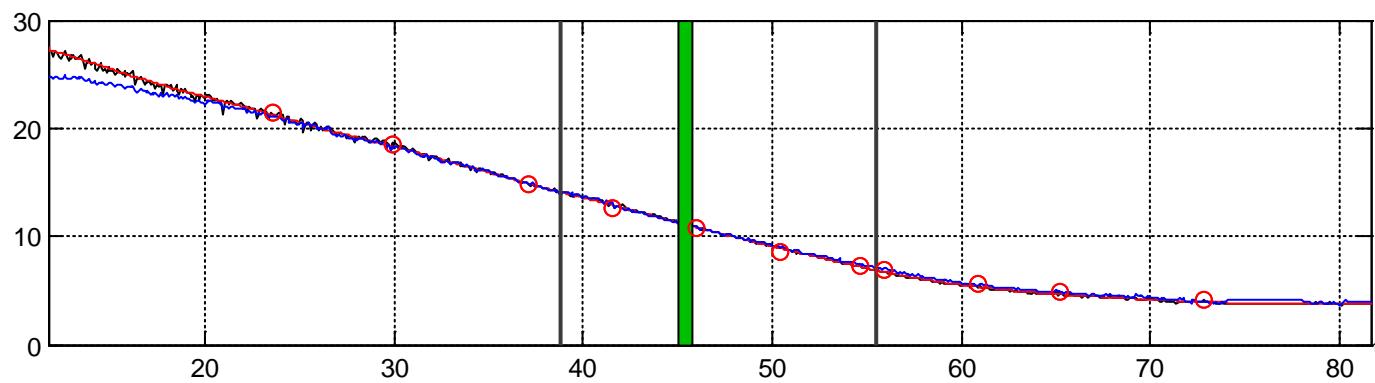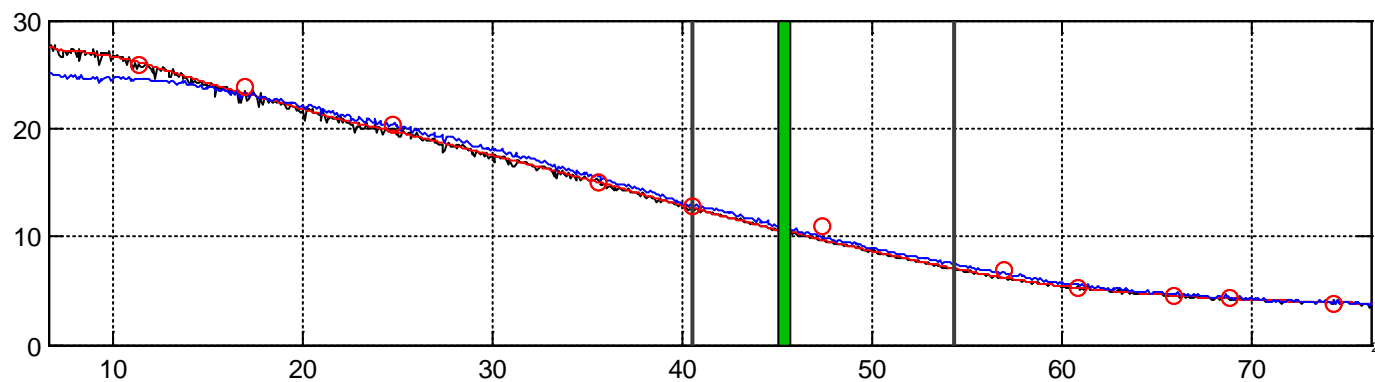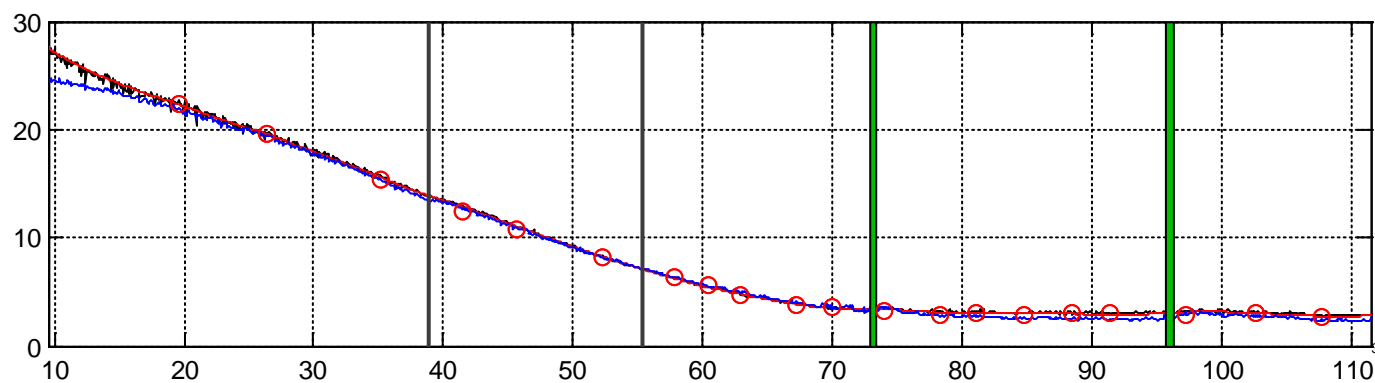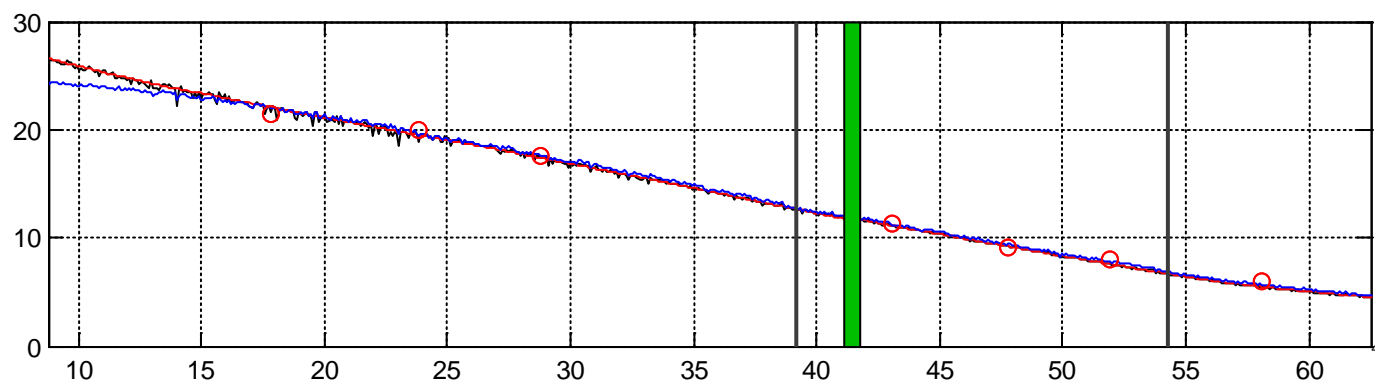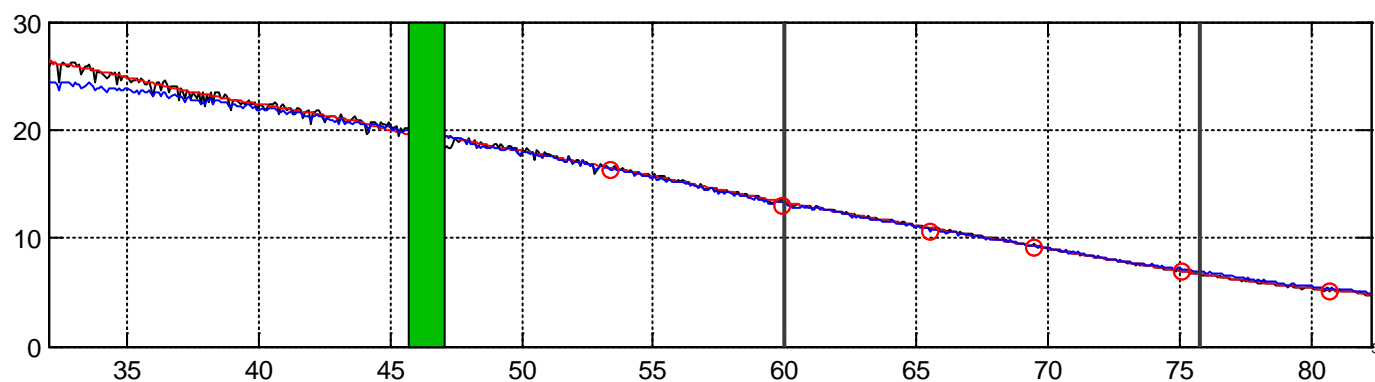

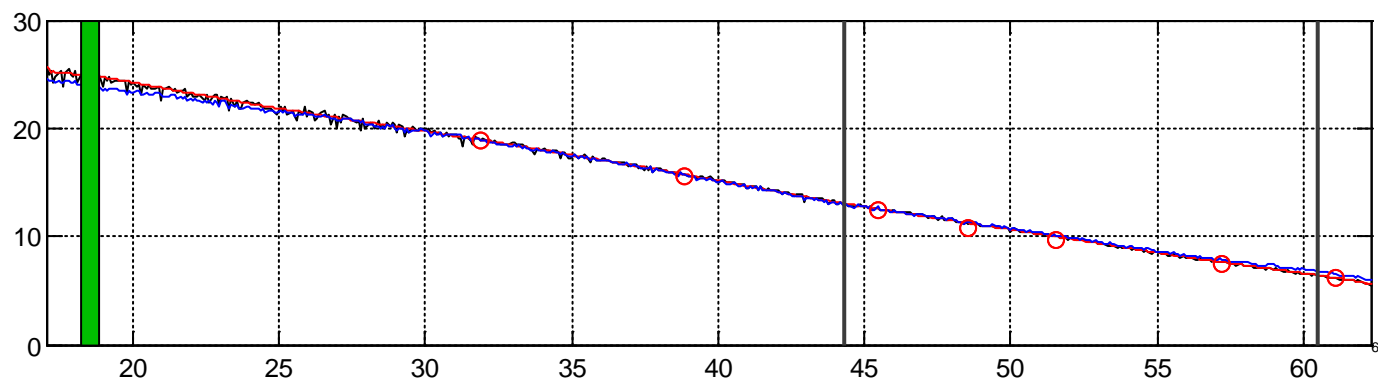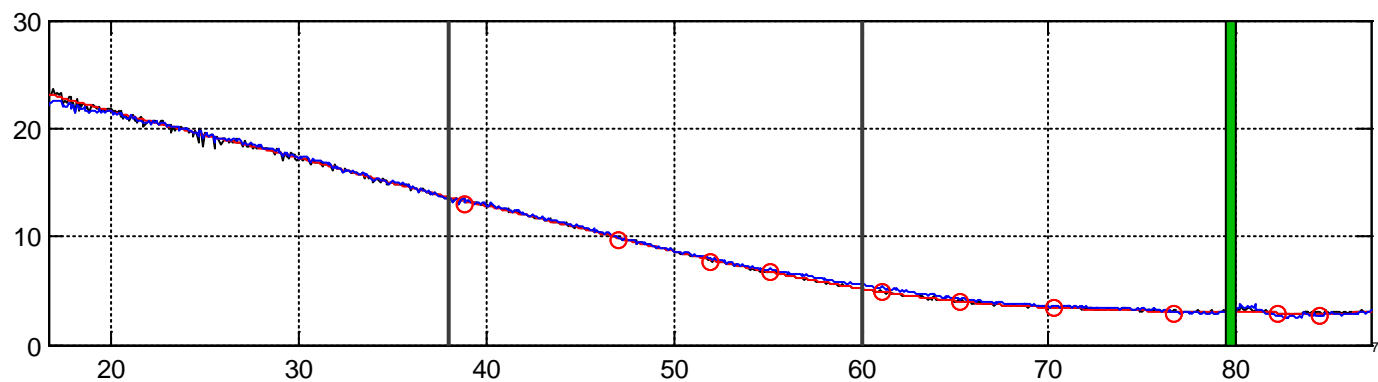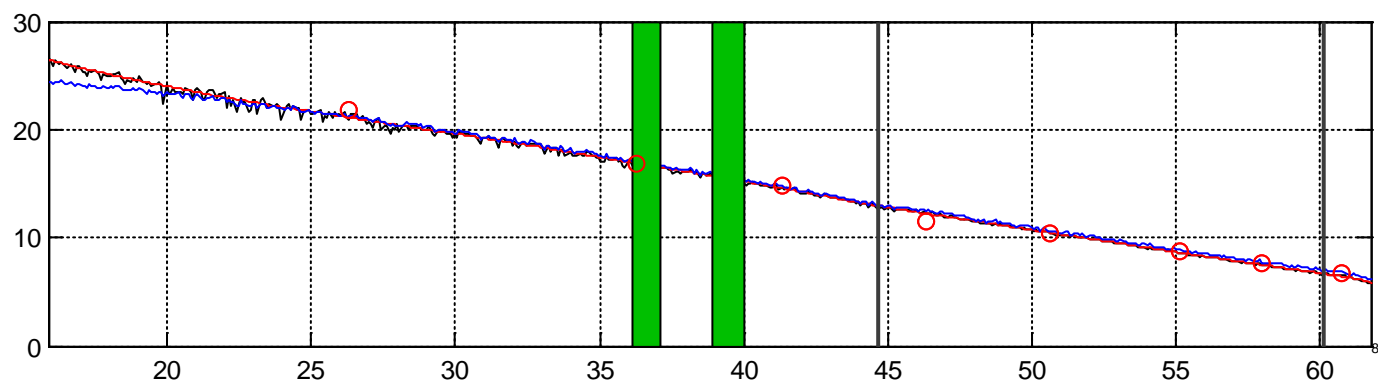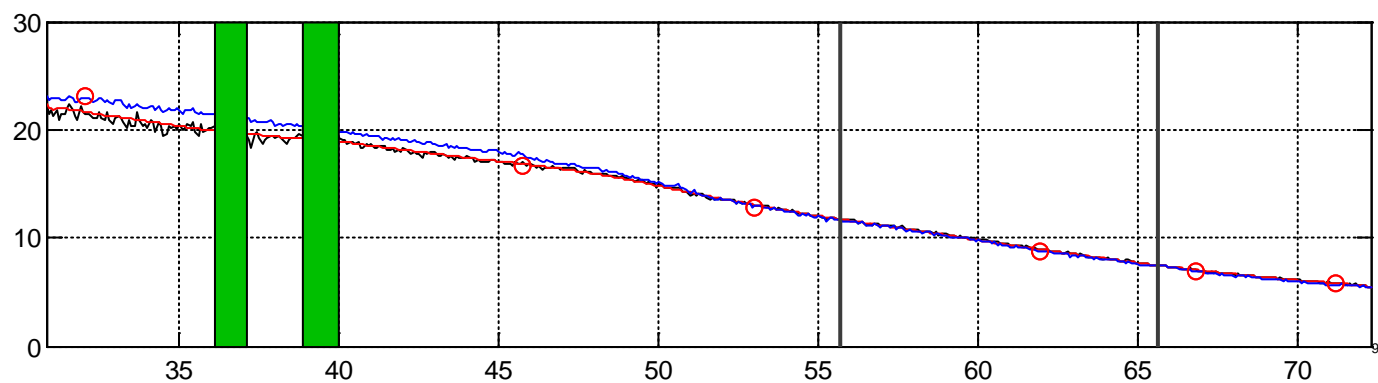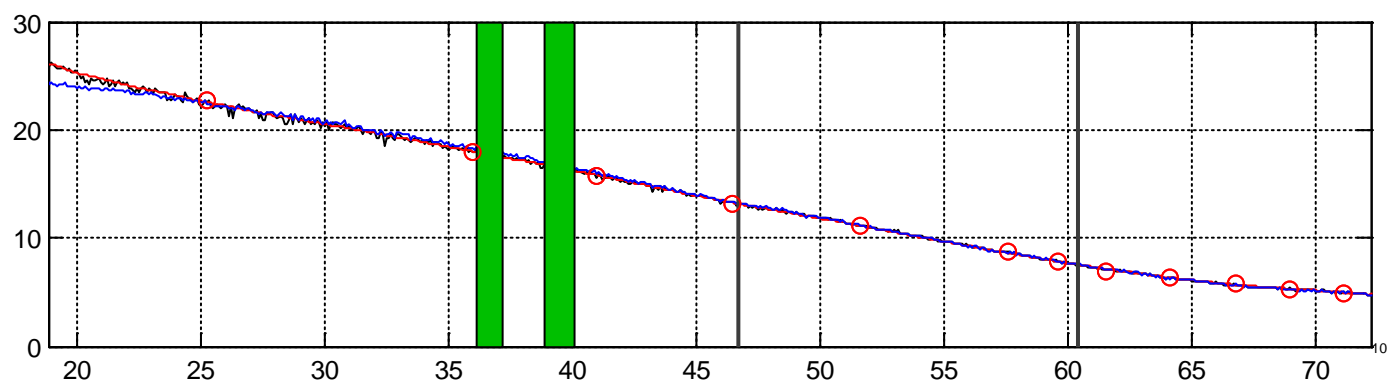

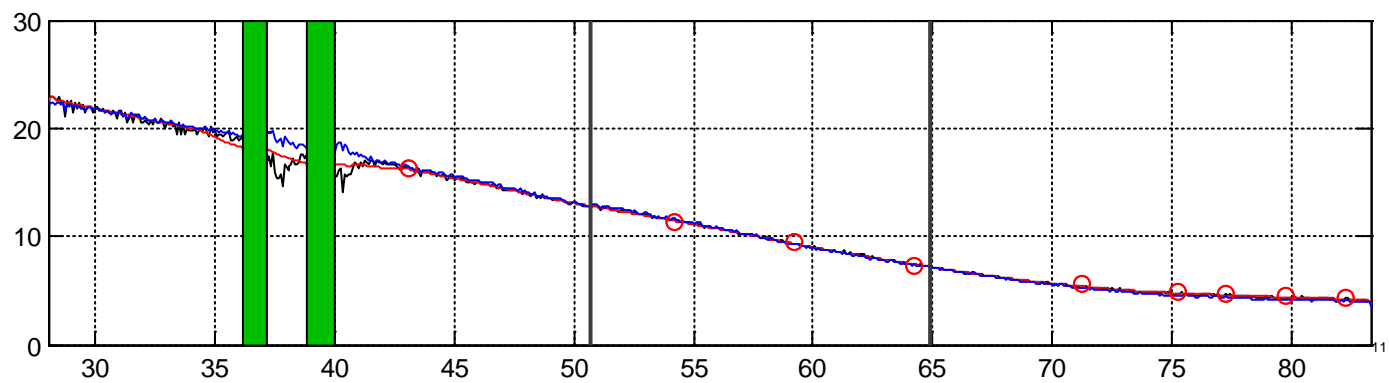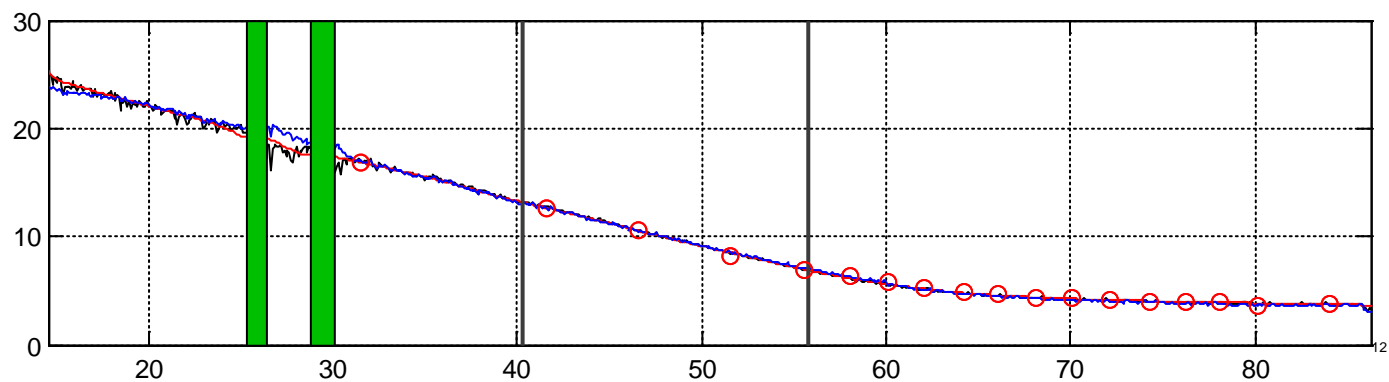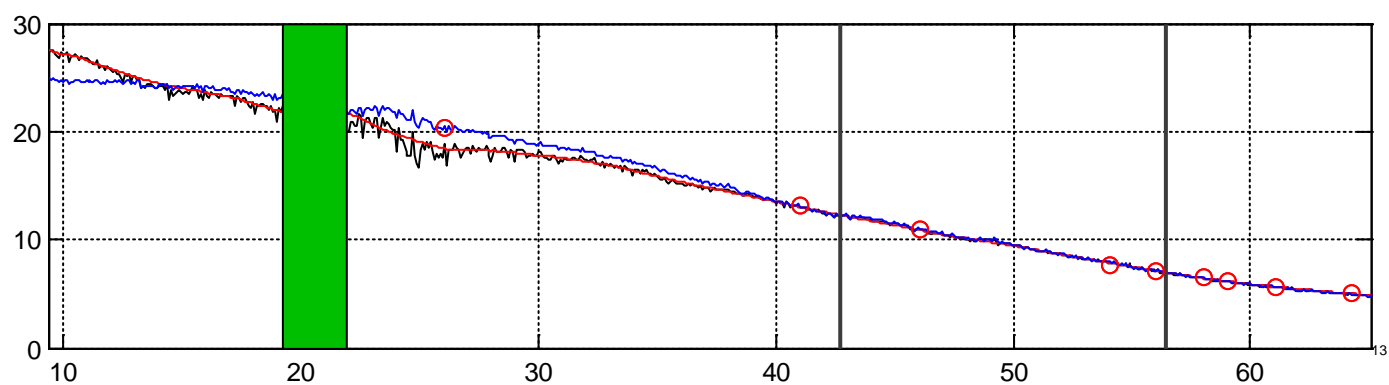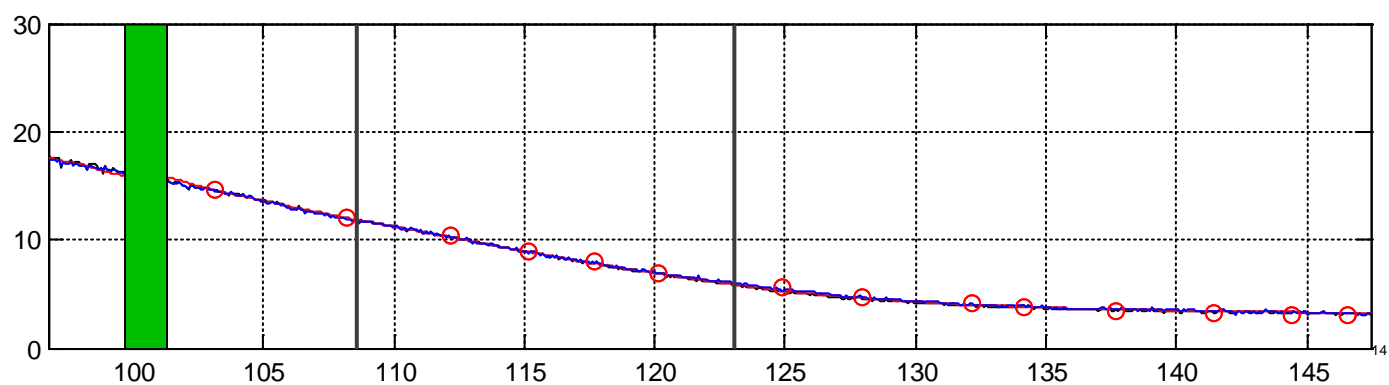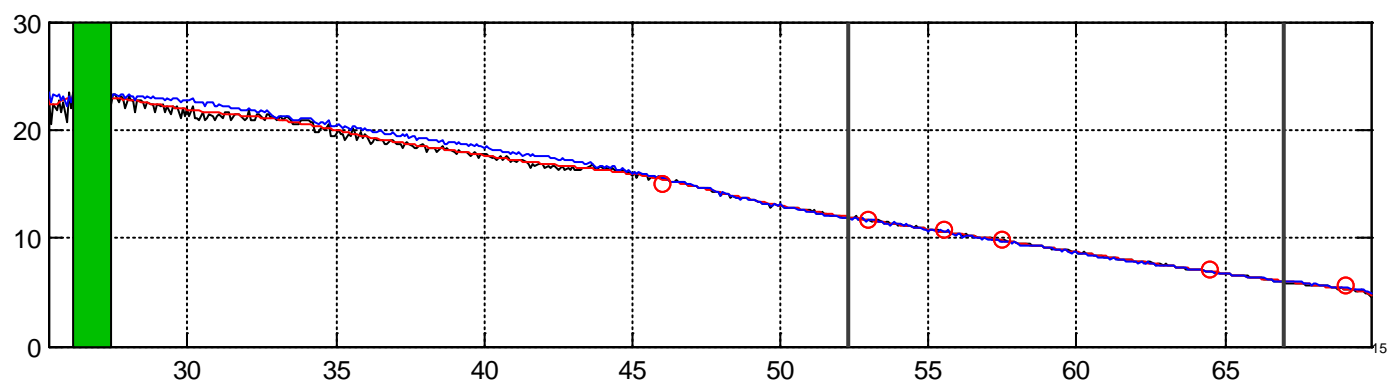

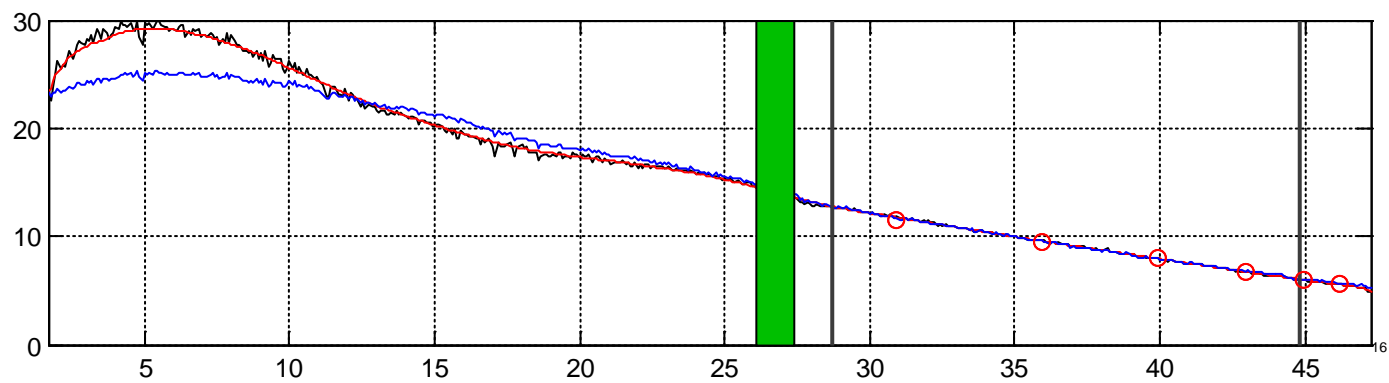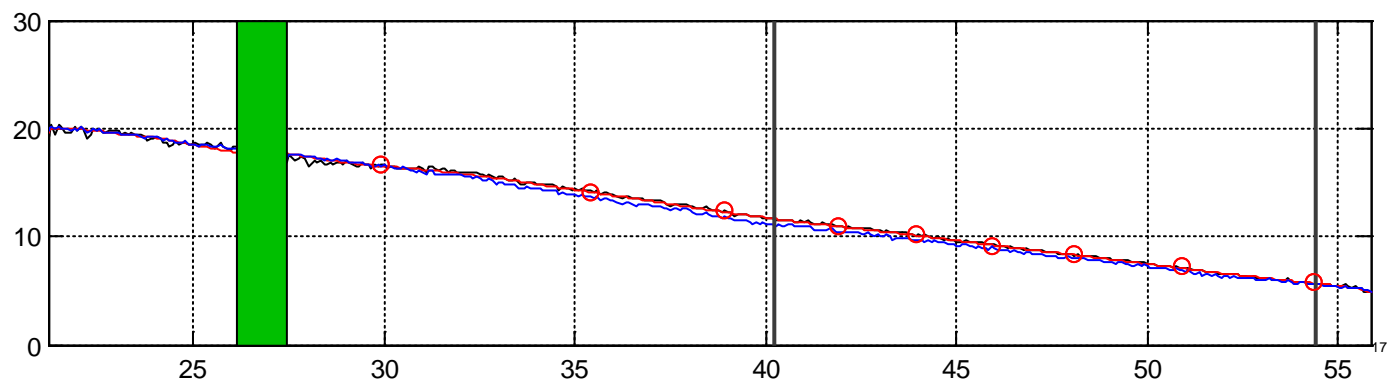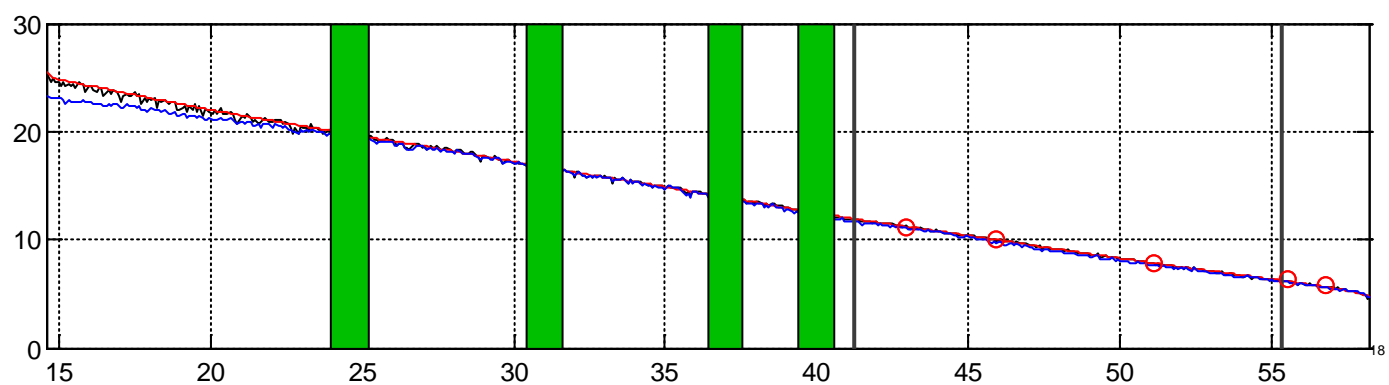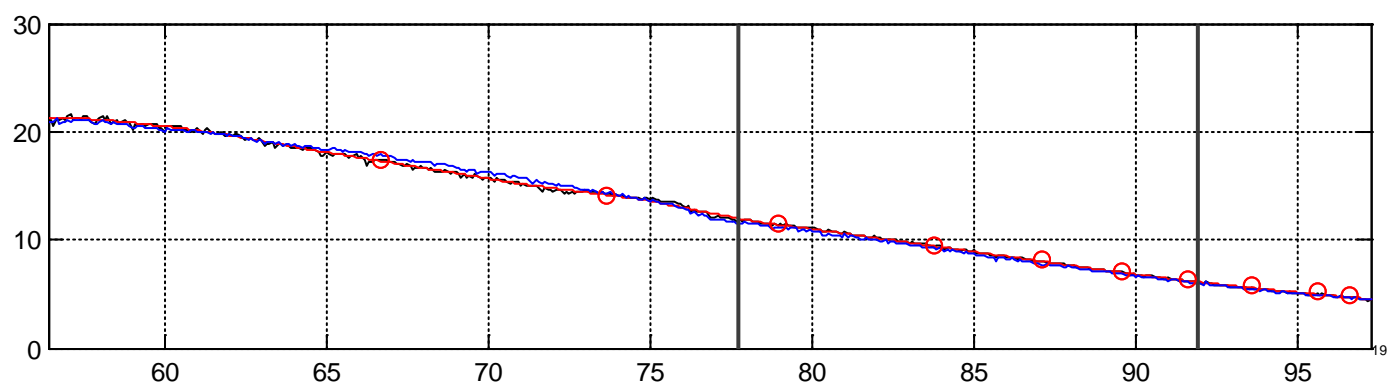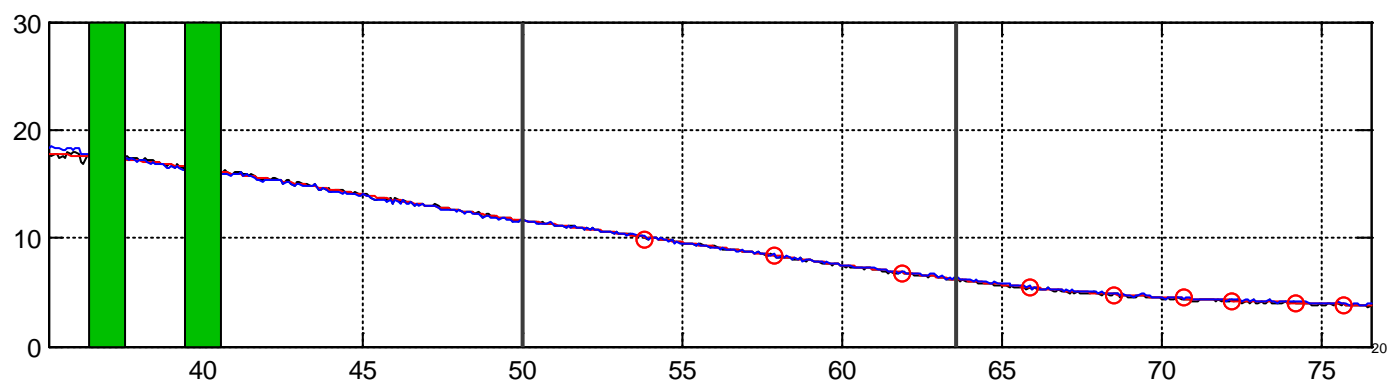

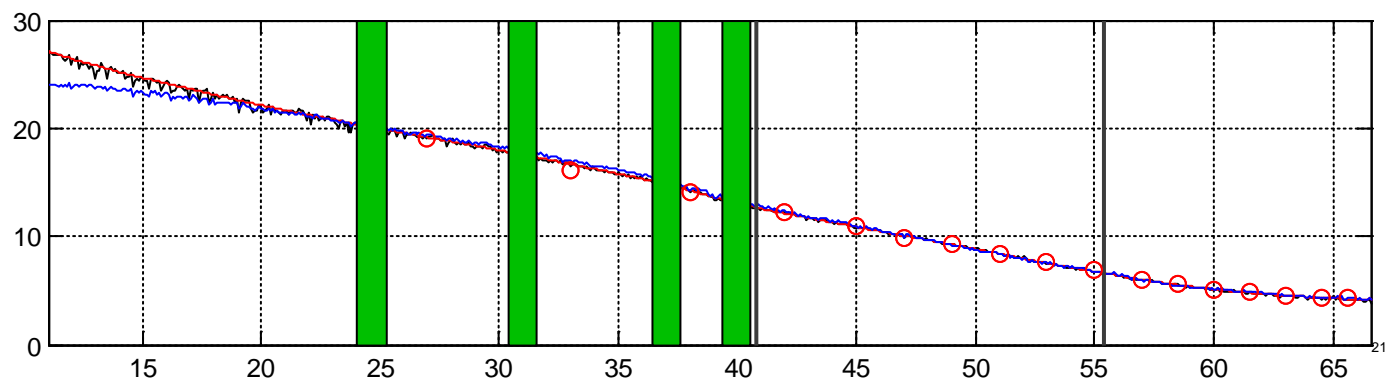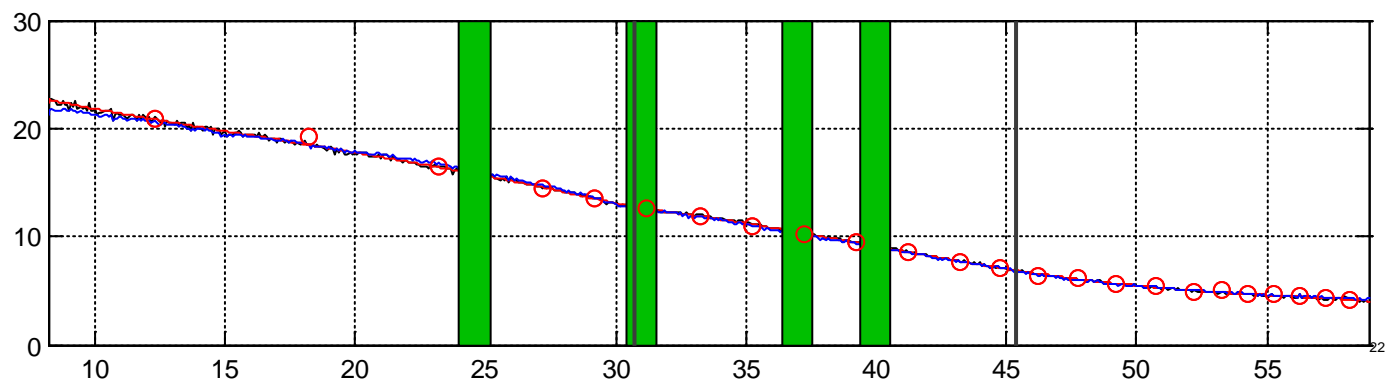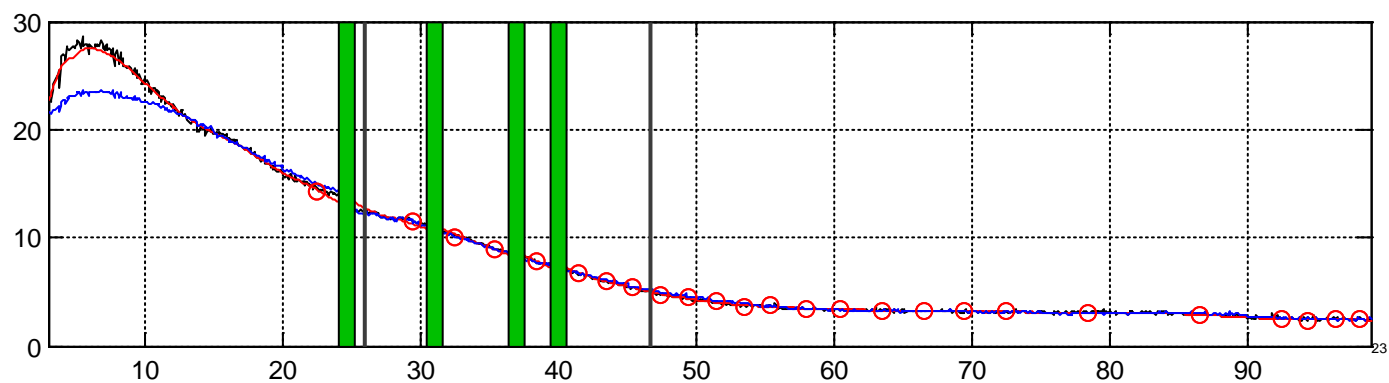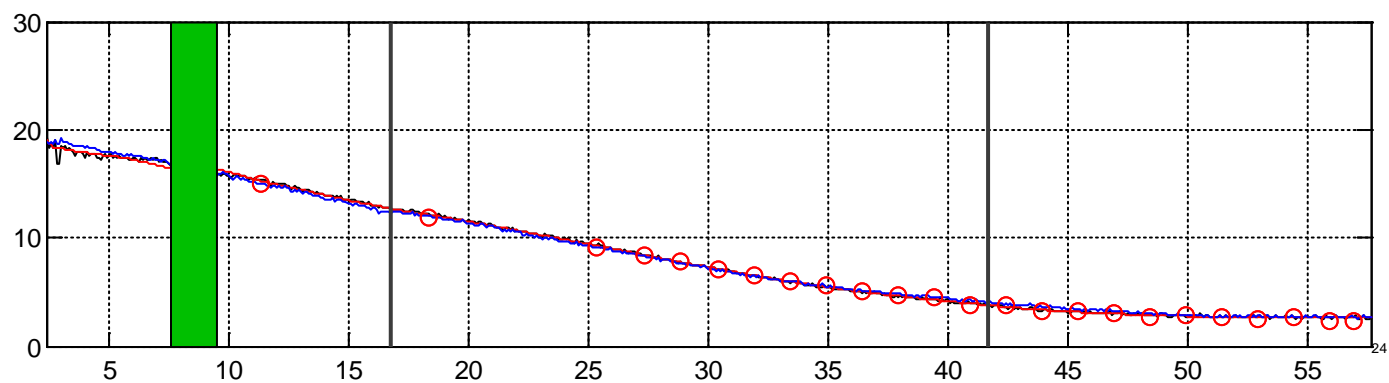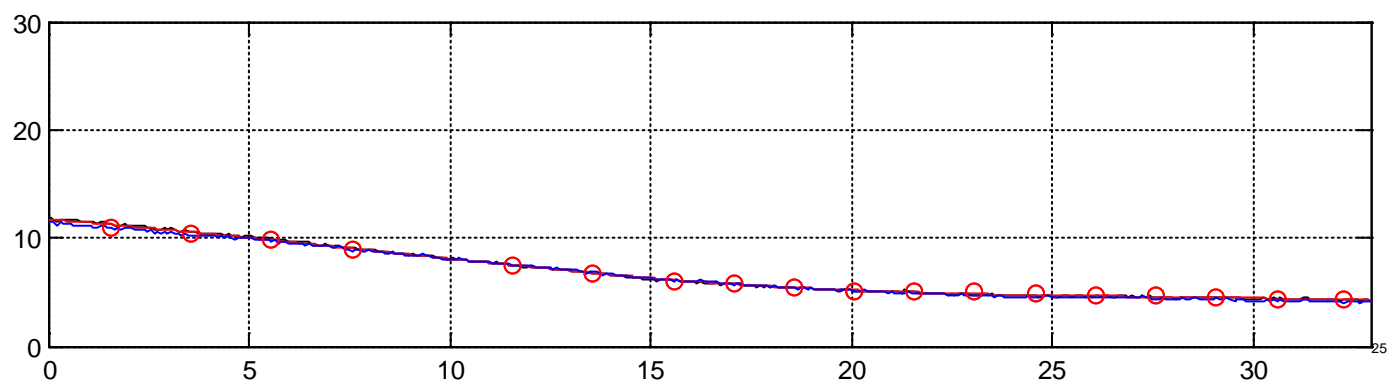

Supplement: Supplementary file 4 [file Image_2.pdf]
